# Sterol Derivatives Specifically Increase Anti-Inflammatory Oxylipin Formation in M2-like Macrophages by LXR-Mediated Induction of 15-LOX

**DOI:** 10.3390/molecules29081745

**Published:** 2024-04-12

**Authors:** Reiichi Ohno, Malwina Mainka, Rebecca Kirchhoff, Nicole M. Hartung, Nils Helge Schebb

**Affiliations:** Chair of Food Chemistry, Faculty of Mathematics and Natural Sciences, University of Wuppertal, Gaußstr. 20, 42119 Wuppertal, Germany

**Keywords:** macrophages, liver X receptor, lipoxygenase, oxylipins, eicosanoids, specialized pro-resolving mediators

## Abstract

The understanding of the role of LXR in the regulation of macrophages during inflammation is emerging. Here, we show that LXR agonist T09 specifically increases 15-LOX abundance in primary human M2 macrophages. In time- and dose-dependent incubations with T09, an increase of 3-fold for ALOX15 and up to 15-fold for 15-LOX-derived oxylipins was observed. In addition, LXR activation has no or moderate effects on the abundance of macrophage marker proteins such as TLR2, TLR4, PPARγ, and IL-1RII, as well as surface markers (CD14, CD86, and CD163). Stimulation of M2-like macrophages with FXR and RXR agonists leads to moderate ALOX15 induction, probably due to side activity on LXR. Finally, desmosterol, 24(*S*),25-Ep cholesterol and 22(*R*)-OH cholesterol were identified as potent endogenous LXR ligands leading to an ALOX15 induction. LXR-mediated ALOX15 regulation is a new link between the two lipid mediator classes sterols, and oxylipins, possibly being an important tool in inflammatory regulation through anti-inflammatory oxylipins.

## 1. Introduction

Inflammation is a protective mechanism against infection or tissue injury. During the course of inflammation, immune cells, such as neutrophils and monocytes, are recruited in several phases and stimulate the formation and release of different cytokines, chemokines, growth factors, and lipid mediators [1,2,3]. In the inflammatory process, monocytes differentiate into macrophages, which have three main tasks: the production of immunomodulators, phagocytosis, and antigen presentation [4,5].

The phagocytosis of cell debris and apoptotic neutrophils as a consequence of acute inflammation serves to restore tissue homeostasis [5,6]. The produced and released immunomodulators, such as interleukin (IL)-1, IL-6, tumor necrosis factor (TNF)-α, interferon (IFN)-α/β, IL-10, IL-12, or IL-18, regulate immune responses, such as stimulation of proliferation of activated natural killer cells or regulation of leukocyte migration from blood to tissue [5,7,8]. In addition, T-cells are attracted by antigen presentation and the release of chemoattractants [5,6].

Primary cell culture of monocyte-derived macrophages allows us to investigate the signaling and regulatory pathways of the innate immune response. Under cell culture conditions, macrophage differentiation is mimicked using different stimuli to investigate the regulation of the arachidonic acid cascade. The (simplified) classification of macrophages is characterized by the expression pattern of characteristic markers, including surface markers as well as pro/anti-inflammatory proteins [9]. Stimulation with granulocyte/macrophage colony-stimulating factor (GM-CSF) leads to the development of a pro-inflammatory state with increased TNF expression and release. After priming with macrophage colony-stimulating factor (M-CSF), the cells exert an anti-inflammatory character with increased IL-10 expression and release. In addition, the polarization of macrophages is induced by stimuli such as cytokines or ligands of the innate immune response, such as bacterial lipopolysaccharide (LPS) [10]. Commonly, stimulation of macrophages with IFNγ and LPS is used to generate classically activated macrophages, i.e., M1-like macrophages [9,11]. The polarization of alternatively activated macrophages is induced by IL-4, IL-10, or IL-13, i.e., M2-like macrophages [9,12]. 

During the inflammatory process, the polarization of macrophages can switch from pro- to anti-inflammatory depending on the inflammatory environment [13,14,15]. A switch of the macrophage phenotype can be initiated by efferocytosis, i.e., the uptake of apoptotic cells [14], which is associated with nuclear receptors such as retinoid X receptors (RXR), PPAR, or liver X receptors (LXR) [16]. Moreover, PPAR is known to be involved in macrophage polarization and inflammatory processes [17]. While inactivation of PPAR or RXR impairs efferocytosis of apoptotic cells, LXR-deficient macrophages are unable to clear apoptotic cells altogether [18,19]. In addition, LXR also drives efferocytosis indirectly by increasing its own expression in an autoregulatory manner [20].

LXR belongs to the family of nuclear receptors involved in the regulation of metabolic homeostasis and inflammation [21]. There are two LXR isoforms that are expressed differently in tissues, despite their high structural similarity (77%). While LXRα is mainly expressed in the liver, kidney, intestine, adipose tissue, or macrophages, LXRβ can be found in all human cells [22,23]. LXR target genes include sterol response element binding protein 1c (SREBP1c), apolipoprotein (apo) E, ATP-binding cassette transporters (ABC)A1, ABCG1, ABCG5, cytochrome P-450 7A1 (CYP7A1), and fatty acid synthase (FAS) [24,25]. As LXR is involved in both cholesterol and lipid metabolism, it could be an important link in the regulation of inflammation by impacting the formation of anti-inflammatory lipid mediators and thus driving the inflammation towards the resolution phase. 

**Figure 1:**
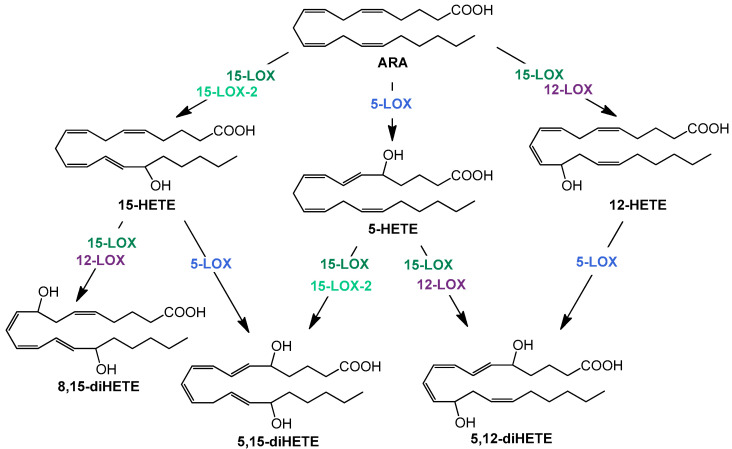
Simplified overview of structures and formation routes of multiple hydroxylated ARA metabolites by the human 5 (blue)-, 12 (purple)-, 15- lipoxygenase (dark green), and 15-LOX-2 (light green).

The resolution phase macrophages produce a variety of anti-inflammatory cytokines and chemokines. In addition, the formation of lipid mediators—oxylipins—plays a decisive role in the course of inflammation. High levels of cyclooxygenase (COX)- and 5-lipoxygenase (LOX)-derived products from arachidonic acid (ARA) are well investigated pro-inflammatory oxylipins, e.g., prostaglandin (PG) E2 and D2 or leukotriene (LT) B4, which are predominantly formed and released at the beginning of the inflammation. In contrast, multi-hydroxylated metabolites of different polyunsaturated fatty acids (PUFA) such as ARA, eicosapentaenoic acid (EPA), and docosahexaenoic acid (DHA) are described to be formed during the resolution phase and have anti-inflammatory properties [26,27]. The formation of multi-hydroxylated oxylipins such as 5,15-diHETE and 5,12-diHETE predominantly involves different lipoxygenases (LOX) interacting with each other (Figure 1) and 15-LOX being a key enzyme [28]. Although there is doubt about the formation, detectability, and receptors of multi-hydroxylated LOX products, so-called specialized pro-resolving mediators (SPM) and SPM bearing three hydroxy groups are not formed in macrophages [29], the anti-inflammatory effect of 15-LOX is undisputed [30].

Recently, we have shown that stimulation of macrophages with the synthetic LXR agonist T0901317 (T09) increases the expression of anti-inflammatory genes, such as ALOX15 (15-LOX gene) [31]. Here, we aim to investigate the LXR-induced influence on the ARA cascade and formation of oxylipins in macrophages, as well as different markers involved in differentiation and polarization. For this, human peripheral blood mononuclear cells (PBMC) left untreated or differentiated into M1- and M2-like macrophages were stimulated with T09, and the formation of oxylipins, protein levels, and presence of surface markers were compared to non-stimulated cells. The specificity of nuclear receptor activation was investigated using FXR agonists and RXR agonists. Finally, sterols were identified as endogenous LXR ligands, elevating 15-LOX abundance and upregulating oxylipin formation. In summary, with the sterol-mediated induction of oxylipins, we demonstrated a new link between these lipid mediator classes in the regulation of M2-like macrophages.

## 2. Results

In the present study, we investigated the effect of LXR activation by different nuclear receptor agonists and endogenous lipids on the induction of ARA cascade enzymes and oxylipin formation, as well as the overall effects on proteins and markers that are involved in the polarization of human primary macrophages.

**Figure 2:**
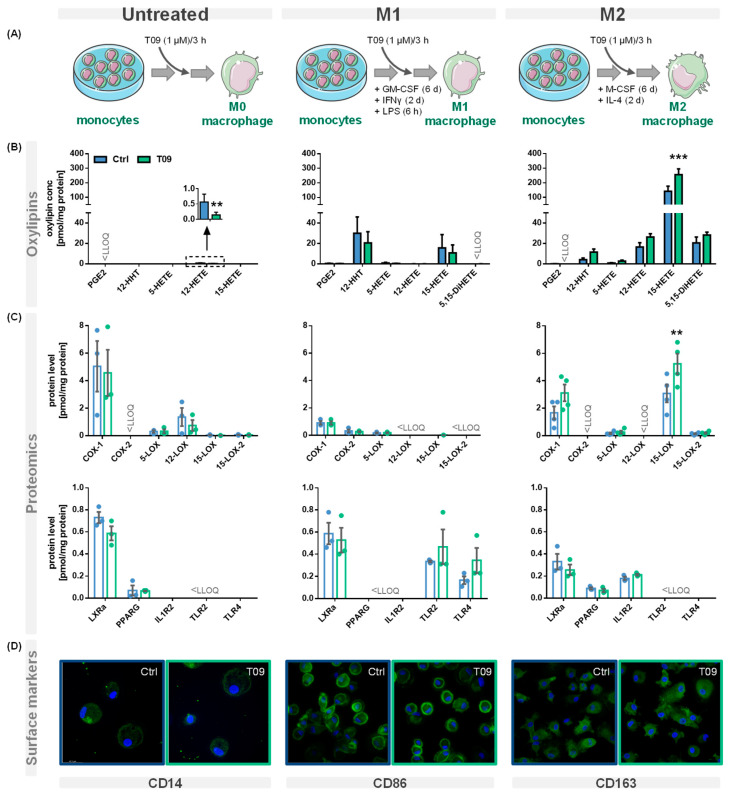
Effect of the LXR agonist T09 on the ARA cascade and differentiation of primary macrophages. (**A**) Primary human blood monocytic cells were treated with 10 ng/mL GM-CSF (M1 type) for 8 days or with M-CSF (M2 type) for 8 days, as well as 10 ng/mL IFNγ (M1 type) or IL-4 (M2 type) for the final 2 days. Additionally, M1 cells were challenged with 100 ng/mL LPS for 6 h. For the untreated macrophages, the adhered monocytes were left untreated for 8 days. The three different macrophage phenotypes were incubated with (blue) or without the synthetic LXR agonist T09 (1 µM; green) for the final three hours. Shown are (**B**) oxylipin concentrations and (**C**) protein levels (mean ± SEM; cells from 3–5 donors). (**D**) The three macrophage phenotypes with or without T09 (1 µM) were immunostained for specific macrophage markers CD14 (untreated), CD86 (M1), or CD163 (M2) using specific mouse anti-human antibodies (green) (magnification 63×). Nuclei were counterstained with Hoechst (blue). The immuno-positivity of the antibodies towards the respective macrophage types is shown in Figure S1. Differences were considered significant at *p*-values < 0.05 (**) or < 0.01 (***) using a two-tailed, unpaired student’s *t*-test.

PBMC were left untreated or differentiated into M1- or M2-like macrophages and stimulated with the synthetic LXR agonist T09 (1 µM) for 3 h (Figure 2A). Oxylipins were analyzed using a LC-MS/MS targeted oxylipin metabolomics method. Figure 2B shows representative oxylipins derived from ARA. The analysis of the protein levels was carried out by LC-MS/MS based targeted proteomics (Section 1 + Tables S1–S3, Supplementary Material). The effect of T09-mediated LXR activation on ARA cascade enzymes, receptors, and other protein levels specific for macrophages is depicted in Figure 2C. The presence of macrophage surface markers reflecting their polarization was analyzed by immunofluorescence staining (Figure 2D). T09 had no effect on the morphology or confluency of untreated M1- and M2-like macrophages.

**Figure 3:**
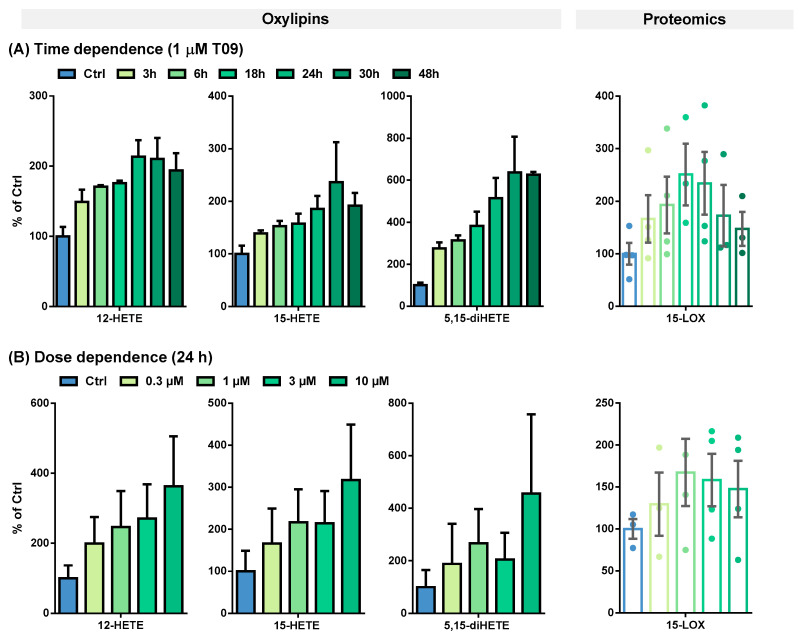
(**A**) Time- and (**B**) dose-dependent T09-induced 15-LOX abundance and activity in primary M2-like macrophages. Shown is the increase of ARA-derived 15-LOX metabolites 12-HETE, 15-HETE, and 5,15-diHETE (**left**) and 15-LOX abundance (**right**). Results are shown as % of Ctrl (mean ± SEM, cells from 3–5 donors).

The T09-induced 15-LOX abundance and activity were further evaluated in a time- and dose-dependent manner (Figure 3 and Table 1).

**Figure 4:**
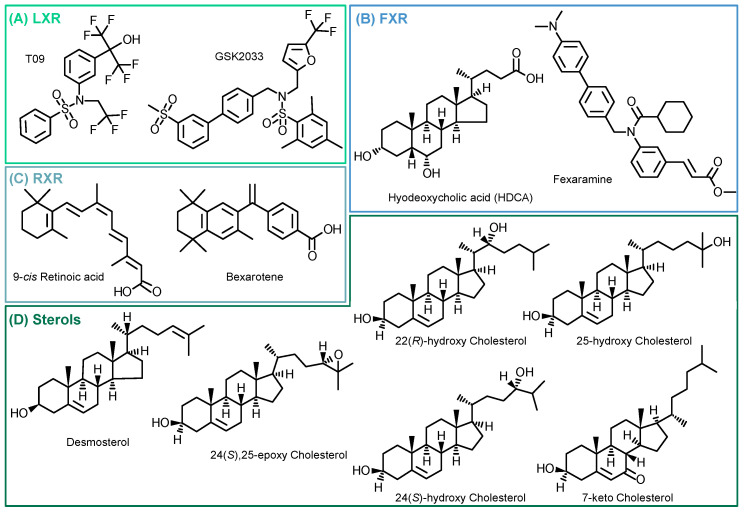
Structures of investigated compounds. (**A**) LXR agonist (T09) and antagonist (GSK2033); (**B**) FXR agonists (Fexaramine and Hyodeoxycholic acid); (**C**) RXR agonists (Bexarotene and 9-*cis* Retinoic acid); and (**D**) tested cholesterol precursor (desmosterol) and oxidized metabolites.

**Figure 5:**
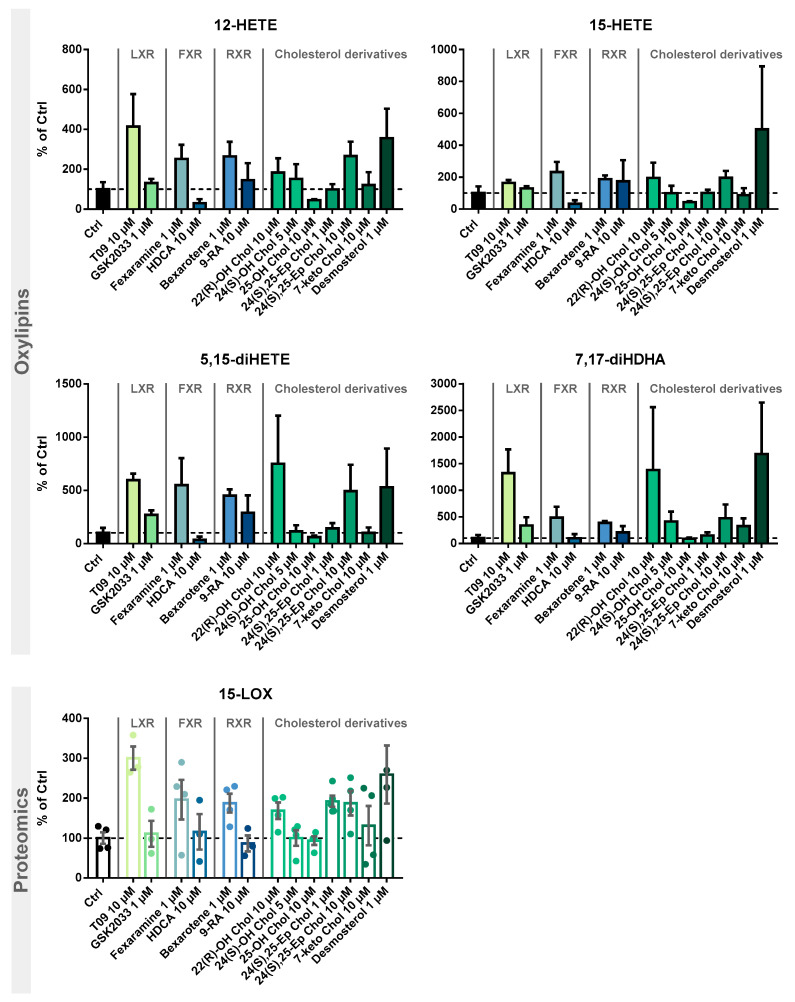
Investigation of 15-LOX induction by LXR, FXR, and RXR agonists and identification of cholesterol precursors and metabolites. M2-like macrophages were incubated with test compounds for 24 h. Shown is the increase of 15-LOX-derived mono-hydroxylated metabolites 12-HETE and 15-HETE (**top**) and multiple-hydroxylated metabolites 5,15-diHETE and 7,17-diHDHA (**middle**), as well as 15-LOX abundance (**bottom**). Results are shown as % of Ctrl (mean ± SEM, cells from 3–5 donors).

Following the evaluation of the optimal incubation conditions, the induction of 15-LOX by other LXR-, FXR-, and RXR-specific compounds as well as cholesterol derivatives (Figure 4) was investigated. The results are shown in Figure 5 and Table S4.

## 3. Discussion

The nuclear receptor LXR is associated with different immune cell functions [32]. We have recently shown that stimulation of M2-like macrophages with LXR agonist T09 leads to overexpression of ALOX15 as well as increased formation of 15-LOX-derived oxylipins [31]. 15-LOX as well as its products, such as 15-HETE or 15-HEPE, are discussed as having anti-inflammatory properties [30,33,34,35].

Here, we investigated the effect of LXR activation on the enzymes of the ARA cascade and oxylipin formation, as well as the effect on markers of human primary macrophages involved in polarization, in detail (Figure 2A). Our experiments demonstrate that in IL-4-stimulated M2-like macrophages, 15-LOX abundance is increased by T09 (Figure 2B,C). The LXR agonist T09 has a specific effect on 15-LOX abundance and its metabolites (15-HETE, 5,15-diHETE). T09 showed no or a low effect on PPARγ, IL-1RII (Figure 2C), and the surface marker CD163 (Figure 2D), which are highly expressed in M2-like macrophages [36,37], and thus this indicates that polarization of M2 macrophages was not influenced by T09-stimulation.

In M1-like macrophages, T09 also has a neglectable effect on the investigated markers involved in the differentiation process (Figure 2C,D). The surface marker CD86, which is highly expressed in M1-like macrophages after IFNγ stimulation [38], remained unchanged. Stimulation with T09 had no effect on the 15-LOX-derived 15-HETE and did not increase ALOX15 abundance in these cells. Stimulation with T09 hardly modulated M1-specific proteins, with a trend towards higher TLR2 and TLR4 amounts and a decrease of COX-2 and its products PGE2 and 12-HHT (Figure 2B,C). 

The untreated macrophages have low levels of PPARγ and the surface marker CD14 remained unchanged after stimulation with T09. Interestingly, incubations with T09 reduced 12-HETE formation (Figure 2B) and 12-LOX protein levels (Figure 2C). However, concentrations of 12-HETE were low and originated from platelets present in cell preparations due to unavoidable platelet contaminations [39]. Again, neither elevated 15-HETE nor changed 15-LOX abundance were observed after T09 incubation in untreated macrophages. 15-LOX-2, which also catalyzes the formation of 15-HETE among other oxylipins, is found in low concentration in untreated cells, and its abundance is not stimulated by T09. While all macrophage phenotypes have comparable levels of LXRα (Figure 2C), only incubations of M2-like macrophages with the LXR agonist T09 specifically increase 15-LOX abundance and its oxylipins. No other major effect of T09 on macrophage markers, namely IL-1RII and PPARγ in M2-like and TLR2 and TLR4 in M1-like macrophages, was observed (Figure 2C). Surface markers CD14, CD86, and CD163 (Figure 2D) were not changed after T09 stimulation, suggesting T09 has no effect on specific surface receptors characteristic of macrophage polarization.

EPA- or DHA-derived mono-hydroxylated oxylipins 12-HEPE, 15-HEPE, 14-HDHA, 7-HDHA, and 17-HDHA were not affected by T09 stimulation (Figure S2, Table 1). Therefore, LXR agonist T09 specifically effects 15-LOX in M2-like macrophages.

This effect on 15-LOX in M2-like macrophages was studied more detailed in terms of the time- and dose-dependent LXR activation by T09 (Figure 3). The highest 15-LOX concentration (2.8-fold) was after a 24-h incubation (Figure 3A, Table 1). An increase in oxylipin concentrations was detected up to 24 or 30 h (Figure 3A, Table 1). Overall, the LXR-induced relative increase in oxylipin formation is more pronounced for multi-hydroxylated oxylipins (3- to 6-fold increase), such as ARA-derived 5,15-diHETE (Figure 3A), 5,12-diHETE, and 8,15-diHETE, EPA-derived 5,15-diHEPE, or DHA-derived 7,17-diHDHA (Figure S3A), than for the ARA-derived mono-hydroxylated oxylipins (2.5-fold increase) (Figure 3A, Table 1) and EPA, or DHA-derived mono-hydroxylated oxylipins (1.5-fold increase) (Figure S3A, Table 1). However, when considering the absolute amount of formed oxylipins, the levels of mono-hydroxylated oxylipins are several times higher: 184 pmol/mg protein for ARA-derived 15-HETE vs. 9.18 pmol/mg protein for 5,15-diHETE, and 45.4 pmol/mg protein for DHA-derived 17-HDHA vs. 10.7 pmol/mg protein for 7,17-diHETE (Table 1).

A strong LXR-induced dose-dependent ALOX15 abundance in M2-like macrophages was observed after 24 h of incubation (Figure 3A). At 1 µM T09, 15-LOX abundance can be maximally increased 2–3-fold; higher concentrations led to no further increase in the abundance of 15-LOX (Figure 3B). The mono-hydroxylated oxylipins show a 2–4-fold higher concentration (Figure 3B and Figure S3B), whereas the concentration of the multi-hydroxylated metabolites is increased up to 15-fold (Figure 3B and Figure S3B). No saturation could be achieved in the dose-dependent stimulation of M2-like cells; higher T09 concentrations were found to be cytotoxic (Figure S5). Nevertheless, incubations with 1 µM T09 reached maximum levels of 15-LOX and oxylipins, higher than all other reports about this enzyme and its products, even compared to IL-4-induced macrophages [34,40].

So far, the presence of the cytokines IL-4 and IL-13 has been required to induce ALOX15 at the mRNA and protein levels in human macrophages in cell culture experiments [34,41]. Even with prolonged incubations with 100 ng/mL LPS for more than 16 h, the 15-LOX abundance at the mRNA as well as protein level remain unchanged, whereas the 15-LOX-derived oxylipins increase by a factor of 1.5–2 [40]. In addition, the concentrations of multi-hydroxylated oxylipins, such as 5,15-diHETE and 7,17-diHDHA, were at most increased by factor 2 [40]. The LXR-mediated effect is stronger than the previously described results on 15-LOX activity in macrophages and thus could be of high biological relevance in the regulation of inflammatory pathways in macrophages. However, other pathways may also be involved in the regulation of 15-LOX abundance and activity.

15-LOX is involved in the regulation of inflammation and is thought to play a protective role in arthritis, promote wound healing and host defense and counteract fibrosis [42,43,44]. This is supported on the one hand by experiments of ALOX15 silencing in various experimental models, which was associated with inflammation and tissue damage [42]. On the other hand, overexpression of human reticulocyte 15-LOX in experimental models protected transgenic animals against atherosclerosis [45].

Anti-inflammatory properties have been described for several of the 15-LOX products. ARA-derived 15-HETE can activate PPARγ and inhibit neutrophil migration, degranulation, and superoxide formation [17,45,46,47,48,49]. 15-HETE can also reduce inflammatory signaling by regulating the TNFα mRNA half-life [50,51]. EPA-derived 15-HEPE is also thought to have anti-inflammatory properties. In transgenic fat1 mice expressing an n3-PUFA generating desaturase, a protective effect was observed after DSS-induced colitis, which was attributed to the high concentrations of 12-HEPE, the major 15-LOX metabolite of EPA in mice. The results were corroborated by silencing of ALOX15 in fat1 transgenic mice, where 12-HEPE concentration was strongly reduced. Moreover, the 15-LOX metabolite 15-HEPE was also reduced in ALOX15-silenced fat1 transgenic mice, and the wild-type mice were protected against the development of DSS-induced colitis after intraperitoneal injections with 15-HEPE [35].

Upon interaction with several LOX enzymes, 5,15-diHETE or 8,15-diHETE can be formed, which are associated with neutrophil and eosinophil chemotactic activity [46,52,53]. EPA-derived 5,15-diHEPE, also known as resolvin E4, has been described to induce efferocytosis of apoptotic neutrophils in human macrophages and senescent red blood cells along with an upregulation of PPARγ gene expression, leading to a resolution of inflammation [54]. In addition, the ALOX15 DHA-derived multi-hydroxylated metabolite 7,17-diHDHA, also called resolvin D5, increases phagocytic activity in neutrophils and macrophages and decreases the formation of pro-inflammatory mediators such as TNFα and NF-κB [55,56]. However, these multi-hydroxylated oxylipins are synthesized in cells and tissues only at low concentrations, and many of the proposed effects and signaling pathways are a matter of discussion [30].

Here, we show that LXR agonist T09 specifically elevates ALOX15 abundance, while the effects on markers and proteins involved in the regulation of inflammation (PPARγ, TLR2, and TLR4 or COX) were minor. This suggests a trend towards a more anti-inflammatory biology in the macrophages due to the increased abundance and activity of 15-LOX. Thus, with the correlation between LXR activation and the formation of oxylipins, we conclude that part of the anti-inflammatory effects of LXR [16,20,31] are mediated by 15-LOX and its products. Given that the effects of 15-LOX modulation are more pronounced compared to the TLR4 ligand LPS, this argues that LXR seems to be a major regulatory pathway in macrophages. However, other pathways might also contribute to this effect, as T09 also has a side activity on other nuclear receptors.

We examined the specificity of the ALOX15 regulation by LXR activation. We analyzed the effects of the related nuclear receptors FXR and RXR (Figure 4) with regard to 15-LOX modulation in M2-like macrophages using specific agonists and antagonists. Incubations with the LXR antagonist GSK2033 did not lead to changes in 15-LOX abundance or the formation of its metabolites (Figure 5), indicating that LXR might not be the only factor involved in ALOX-15 regulation.

Following treatment of M2-like cells with the FXR agonist hyodeoxycholic acid (HDCA) [57], the 15-LOX abundance remains unchanged. The FXR agonist fexaramine leads to a 2-fold increase in 15-LOX abundance (Figure 5 Proteomics), and the corresponding oxylipin concentrations are also increased by 2 to 5-fold (Figure 5 Oxylipins). However, due to the rather nonspecific binding pocket [58,59] of the nuclear receptors, the binding of ligands overlaps. Thus, the effect of fexaramine could be caused by a side activity on LXR, though it was previously described that fexaramine cannot activate LXR [60]. Consistently, it has been shown that the RXR agonists bexarotene and 9-*cis* retinoic acid (9-RA) have agonistic activity toward LXR [61], and thus, bexarotene increased 15-LOX abundance 2-fold (Figure 5 Proteomics) and its products 2- to 5-fold (Figure 5 Oxylipins). These results also might indicate the involvement of other nuclear receptors or an off-target effect of the substances on LXR. 9-RA showed lower effects on 15-LOX: the 15-LOX concentrations apparently remained unchanged while 12-HETE and 5,15-diHETE formation increased (Figure 5).

Finally, we searched for endogenous ligands eliciting the LXR-mediated effects on the ARA cascade in M2-like macrophages (Figure 4D). Oxysterols and metabolites from cholesterol biosynthesis have been previously described as LXR activators [22,62,63,64].

Treatment with the autoxidatively formed 7-keto cholesterol leads to a moderate increase in 15-LOX abundance and activity (Figure 5). The 24(*S*),25-Ep cholesterol elevates 15-LOX products in a concentration-independent manner (2-fold) (Figure 5 Oxylipins), whereas only incubations with 10 µM 24(*S*),25-Ep cholesterol increase mono-hydroxylated oxylipins 3-fold and multi-hydroxylated oxylipins 5-fold (Figure 5 Oxylipins). For 24(*S*)-OH cholesterol and 25-OH cholesterol, a weaker effect was observed as they moderately increased LOX products, while no difference in 15-LOX abundance was observed (Figure 5 Proteomics). For 24(*S*)-OH cholesterol, a trend towards increased 15-LOX-derived oxylipin concentrations (up to 3-fold) is observed (Figure 5 Oxylipins). In incubations with 25-OH cholesterol, the oxylipin concentrations were reduced by half (Figure 5 Oxylipins). 22(*R*)-OH cholesterol has the strongest effect on 15-LOX activity of all the oxysterols studied. While the mono-hydroxylated oxylipins are increased 2-fold, the increase of the multi-hydroxylated oxylipins is up to 15-fold (Figure 5 Oxylipins), together with a 2-fold increase in 15-LOX abundance.

The activity of oxysterols on ALOX15 in M2-like macrophages is consistent with ligand binding affinity to the LXR [21,63]. In structure/activity studies, it was shown that the position of the functional group is of great importance, the essential positions being C-22 and C-24 [63,65]. In our experiments, we were also able to correlate the effect strength with the position of the functional group. Thus, when incubated with 22(*R*)-OH cholesterol and 24(*S*),25-Ep cholesterol, we observed the strongest effect on 15-LOX abundance and activity, whereas with 25-OH cholesterol, hardly any changes were observed. Our results are consistent with the effects described, where a significant LXR activation is observed for 22(*R*)-OH cholesterol > 24(*S*)-OH cholesterol > 25-OH cholesterol, whereas 25-OH cholesterol has little to no effect [21,63,64,66]. Other significantly important ligands are described to be 24(*S*),25-Ep cholesterol, 20(*S*)-OH cholesterol, and 27-OH cholesterol [63,64,67]. Of note, 24(*S*),25-Ep cholesterol is not formed from cholesterol like other oxysterols but is a by-product of cholesterol biosynthesis (mevalonate pathway) [63,67,68]. In addition, the central intermediate from cholesterol biosynthesis, desmosterol, caused strong effects on 15-LOX. Following stimulation with desmosterol, 15-LOX is overexpressed almost 3-fold, which is comparable to the effect of T09 on 15-LOX abundance (Figure 5 Proteomics). 15-LOX activity in incubations with desmosterol and T09 is also comparable, with the mono-hydroxylated oxylipins also being increased 4-fold and the multi-hydroxylated oxylipins up to 16-fold (Figure 5 Oxylipins). Thus, we have found active endogenous LXR ligands, namely desmosterol, 22(*R*)-OH cholesterol and 24(*S*),25-Ep cholesterol, that are able to massively increase 15-LOX abundance and activity through LXR.

Oxysterols occur in mammalian tissues in micromolar concentrations and circulate in only nanomolar concentrations [63,67,69]. Elevated (circulating) oxysterol levels are associated with pathological structures, such as foam cells or atherosclerotic lesions [32,67,70,71]. High 24(*S*)-OH cholesterol plasma levels are associated with Alzheimer’s disease [67,72,73]. Desmosterol can be found accumulated mainly in macrophage foam cells and atherosclerotic plaques, where it regulates via LXR the activation of genes involved in cholesterol efflux [74]. Due to the induction of ALOX15 abundance and the elevated formation of anti-inflammatory oxylipins, desmosterol might also be involved in the reduction of atherosclerotic lesions via LXR activation. 22(*R*)-OH cholesterol, 24(*S*)-OH cholesterol, and 24(*S*),25-Ep cholesterol have been shown to inhibit inflammatory signaling in cell culture models [75]. As many oxysterols and metabolites of cholesterol biosynthesis are involved in the regulation of inflammatory and immune responses [16,76,77,78], this study of the effect of cholesterol derivatives on macrophages is of great importance to better understand the interactions between sterol metabolism and LXR-mediated lipid mediators. The induction of ALOX15 in macrophages is associated with anti-inflammatory properties [45]. Our results show that LXR upregulation may be related to the modulation of 15-LOX and the formation of anti-inflammatory oxylipins. Thus, the observed relationship between LXR and oxylipin formation activated by endogenous sterols may represent an important feedback regulation in macrophages, where sterols indirectly modulate 15-LOX through their binding to LXR, initiating inflammatory resolution. 

Limitations: As with all mechanistic cell culture studies using cell model systems and chemical probes, our study has limitations: The primary macrophages used, derived from different subjects, showed different (basal) levels of 15-LOX abundance and activity, which could be a confounder in the observed regulation by LXR agonists. Macrophages derived from healthy donors from a blood donation center, but no detailed information about their health status, sex, age, or medications were available. These factors might also contribute to the regulation of 15-LOX. Regarding the chemical probes, it should be noted that one agonist and one antagonist of LXR and no antagonists of FXR and RXR could be tested in the study, and they may elicit non-specific effects at the tested concentrations.

## 4. Materials and Methods

### 4.1. Chemicals

Human AB plasma was obtained from the blood donor service of the University Hospital of Düsseldorf (Düsseldorf, Germany). Lymphocyte separation medium 1077 was from PromoCell (Heidelberg, Germany). Recombinant human colony stimulating factors M-CSF and GM-CSF, IFNγ, and IL-4 produced in *Escherichia coli* were purchased from PeproTech Germany (Hamburg, Germany). RPMI 1640 cell culture medium, L-glutamine and penicillin/streptomycin (5.000 units penicillin and 5 mg streptomycin/mL), lipopolysaccharide (LPS) from *E. coli* (0111:B4, product number: L2630), dextran from *Leuconostoc* spp. (molecular weight 450,000–650,000), copper sulfate pentahydrate, iodoacetamide, dimethylsulfoxide (DMSO), and desmosterol were obtained from Sigma (Schnellendorf, Germany). Trypsin (>6000 U/g, from porcine pancreas), protease-inhibitor mix M (AEBSF, Aprotinin, Bestatin, E-64, Leupeptin, Pepstatin A), and resazurin were purchased from SERVA Electrophoresis GmbH (Heidelberg, Germany). Ammonium hydrogen carbonate, sodium deoxycholate, urea, and formaldehyde were from Carl Roth (Karlsruhe, Germany).

Acetonitrile, methanol (LC-MS grade), acetone (HPLC grade), acetic acid (Optima LC-MS grade), and BCA reagent A were purchased from Fisher Scientific (Schwerte, Germany). Ethyl acetate (HPLC grade) and n-hexane (HPLC grade) were bought from VWR (Darmstadt, Germany). The ultra-pure water with a conductivity of >18 MΩ·cm was generated by the Barnstead Genpure Pro system from Thermo Fisher Scientific (Langenselbold, Germany). Oxylipin standards and deuterated oxylipin standards used as internal standards and tested compounds (T0901317 (T09), 22(*R*)-OH cholesterol, 24(*S*)-OH cholesterol, 25-OH cholesterol, 24(*S*),25-Ep cholesterol, 7-keto cholesterol, GSK2033, fexaramine, hyodeoxycholic acid, bexarotene, and 9-*cis* retinoic acid) were purchased from Cayman Chemical (local distributor Biomol, Hamburg, Germany). Unlabeled and heavy labeled (lys: uniformly labeled (U)-^13^C_6_; U-^15^N_2_; arg: U-^13^C_6_; U-^15^N_4_) peptide standards were purchased from JPT Peptides (Berlin, Germany).

### 4.2. Cultivation of Macrophages from Human PBMC

Primary human macrophages were purified and differentiated as described [40]. Buffy coats were obtained from the generation of erythrocyte concentrates from the blood donor services at the University Hospital of Düsseldorf, Germany, with the informed consent of healthy human subjects. The study was approved by the Ethical Committee of the University of Wuppertal. In brief, primary human blood monocytic cells (PBMC) were isolated from fresh buffy coats by dextran (5%) sedimentation for 45 min. The supernatant was layered on lymphocyte separation medium and centrifuged for 10 min at 1000× *g* without deceleration. PBMC sedimented on top of the lymphocyte separation medium was collected. Following washing with PBS twice, cell pellets were resuspended in RPMI 1640 medium supplemented with 1% L-glutamine and 1% penicillin/streptomycin (P/S). Cell suspensions were transferred to petri dishes and incubated for 1 h (37 °C; 5% CO_2_; humidified atmosphere). Afterwards, dishes were washed twice with RPMI 1640 medium supplemented with 1% L-glutamine and 1% P/S to remove non-adherent cells and layered with RPMI 1640 growth medium, which contains 2 g/L glucose and is supplemented with 1% L-glutamine, 1% P/S, and 5% heat-inactivated human AB plasma. The monocytes were differentiated into different macrophage phenotypes for 8 days. For the M1-like phenotype, the growth medium was supplemented with 10 ng/mL GM-CSF, and for the M2-like phenotype, with 10 ng/mL M-CSF. The growth medium was refreshed every other day. In addition, for the last 48 h the cells were incubated with 10 ng/mL IFNγ (M1) or 10 ng/mL IL-4 (M2). Six hours before the harvest, the M1-like cells were additionally stimulated with 100 ng/mL LPS. For the untreated phenotype, cells were cultivated in growth medium without the addition of cytokines. 

The differentiated macrophages were treated with test compounds at different time points and concentrations. 

The cytotoxic effect of the test substances was investigated by means of the resazurin (alamar blue) assay [79], and only non-toxic concentrations were used (Figure S5). 

The cells were harvested using the cold shock method by washing with PBS and incubating in ice cold PBS/EDTA (20 min/4 °C). The cells were collected by scraping, centrifugation, and pelleting followed by washing with PBS containing protease inhibitors. The harvested primary macrophage pellets were frozen at −80 °C until use.

### 4.3. Analysis of Oxylipins and Protein Levels by LC-MS/MS

The analysis of oxylipins and protein levels was carried out from the same cell pellet. Cell pellets were resuspended in PBS containing an antioxidant/inhibitor mixture (0.2 mg/mL BHT, 100 µM indomethacin, 100 µM *trans*-4-(-4-(3-adamantane-1-yl-ureido-9-cyclohexyloxy)-benzoic acid (t-AUCB) in MeOH) and sonicated [80,81]. The protein content was determined by the bicinchoninic acid (BCA) assay. Following addition of 10 µL of the oxylipin internal standards (^2^H_4_-PGE_2_, ^2^H_4_-PGD_2_, ^2^H_4_-TxB_2_, ^2^H_4_-13,14-dihydro-15-keto PGE_2_, ^2^H_4_-15-deoxy-^Δ12,14^-PGJ_2_, ^2^H_4_-6-keto-PGF_1α_, ^2^H_4_-8-*iso*-PGF_2α_, ^2^H_4_-PGE_2_, ^2^H_4_-PGD_2_, ^2^H_4_-TxB_2_, ^2^H_4_-PGF_2α_, ^2^H_5_-RvD2, ^2^H_11_-8,12-*iso*-iPF_2α_-VI, ^2^H_5_-LxA_4_, ^2^H_5_-RvD1, ^2^H_4_-PGB_2_, ^2^H_4_-LTB_4_, ^2^H_4_-9,10-DiHOME, ^2^H_11_-11,12-DiHETrE, ^2^H_6_-20-HETE, ^2^H_4_-13-HODE, ^2^H_4_-9-HODE, ^2^H_8_-15-HETE, ^2^H_3_-13-oxoODE, ^2^H_8_-12-HETE, ^2^H_8_-5-HETE, ^2^H_4_-12(13)-EpOME, ^2^H_11_-14(15)-EpETrE, ^2^H_7_-5-oxoETE, ^2^H_11_-8(9)-EpETrE, each 100 nM in MeOH) and protein precipitation in methanol (−80 °C; 30 min), the supernatant was used for the oxylipin analysis whereas the protein pellet was frozen at −80 °C for a later protein level analysis [39].

The oxylipin analysis was carried out as described [80,81]. In brief, the oxylipins were extracted from the supernatants using Bond Elut Certify II SPE cartridges (200 mg, 3 mL, Agilent, Waldbronn, Germany). Following elution of the oxylipins using ethyl acetate/n hexane/acetic acid (75/25/1, *v*/*v*/*v*), samples were evaporated (vacuum concentrator, 30 °C, 1 mbar; Christ, Osterode, Germany), and the residue was reconstituted in 50 µL internal standard 2 (1-(1-(ethylsulfonyl)piperidin-4-yl)-3-(4-(trifluoromethoxy)-phenyl)-urea, 12-(3-adamantan-1-yl-ureido)-dodecanoic acid, 12-oxo-phytodienoic acid, and aleuritic acid) [80,81]. Oxylipins were analyzed using an 1290 Infinity II System (Agilent) coupled to a 5500 QTRAP instrument (Sciex, Darmstadt, Germany) in ESI(−)-mode operated in scheduled selected reaction monitoring [82].

The analysis of the protein levels was carried out as described [39,83]. In brief, the protein pellet was dissolved in 5% (*w*/*v*) sodium deoxycholate with protease inhibitor (100/1; *v*/*v*), precipitated with ice-cold acetone (4 volumes), and centrifuged (4 °C, 15,000× *g*, 20 min). Following subsequent incubations with 200 mM dithiothreitol (in 50 mM NH_4_HCO_3_), 200 mM iodoacetamide (in 50 mM NH_4_HCO_3_), and again 200 mM dithiothreitol, the proteins were digested overnight (15 h) using 100 µg/mL trypsin in 50 mM acetic acid (trypsin-to-protein ratio of 1:50). The reaction was stopped by acidification with concentrated acetic acid (pH 3–4). Following the addition of internal standards (heavy labeled peptides with sequences corresponding to each analyte peptide), the samples were extracted using Strata-X SPE 33 µm Polymeric Reversed Phase cartridges (100 mg/3 mL, Phenomenex LTD, Aschaffenburg, Germany). The peptides were eluted with 70% acetonitrile/0.1% acetic acid, evaporated, and finally reconstituted in 15% acetonitrile/0.1% acetic acid. The peptides were analyzed on an 1290 Infinity II System (Agilent) coupled to a 6500+ QTRAP instrument (Sciex) in ESI(+)-mode operated in scheduled selected reaction monitoring mode [83].

MS data analysis was performed with the software MultiQuant^TM^ 3.0.2 (Sciex) using a Gaussian smooth width of 1 and the MQ4 integration algorithm. Concentrations were calculated via external calibration using internal standards based on the ratio of the analyte and internal standard areas. The quantified peptide/protein and oxylipin concentrations were normalized to the absolute protein content determined via the BCA assay.

### 4.4. Immunofluorescence Labelling

PBMC were seeded in 24-well plates on coverslips (Sarstedt, Nümbrecht, Germany) and cultivated as described above. For labeling, polarized macrophages were fixed in 4% formaldehyde in PBS for 15 min at 37 °C. Following washing with TBS the cells were permeabilized and blocked with TBS, containing 0.3% Tween 20 and 1% bovine serum albumin (BSA) for 30 min at 30 °C. The primary macrophages were then incubated overnight with the primary antibodies 1:100 diluted in TBS/0.3% Tween 20/1% BSA against CD14, CD86, and CD163 (all mouse anti-human; Bio-Rad Laboratories, Feldkirchen, Germany). The coverslips were washed extensively with TBS/0.3% Tween 20 and then incubated with FITC-conjugated secondary antibody (1:300 in TBS/0.3% Tween 20/1% BSA; goat anti-mouse IgG Alexa Fluor 488, Thermo Scientific) for 45 min at 30 °C and again extensively washed with TBS/0.3% Tween 20. The nuclei were stained with 10 µg/mL Hoechst 33258 (Sigma) for 45 s and finally the coverslips were observed using a fluorescence microscope (Leica DM6 B, Wetzlar, Germany) [84].

### 4.5. Statistical Analysis

Statistical analysis was performed using GraphPad Prism (GraphPad Software Inc. version 6.01, San Diego, CA, USA). Data are presented as mean ± standard error of mean (SEM). Statistical analyses were performed by a two-tailed, unpaired student’s *t*-test. Differences were considered significant at *p*-values < 0.05 or 0.01.

## 5. Conclusions

We demonstrate that LXR activation by T09 exerts dramatic effects on the ARA cascade, specifically in M2-like macrophages, by increasing the abundance of 15-LOX and increasing the formation of its products. No or only a moderate effect of T09 was observed on untreated and M1-like macrophages, as well as surface markers and PPARγ, IL-1RII, TLR2, and TLR4 abundance.

The investigated agonists of FXR and RXR only had a moderate effect on 15-LOX abundance and activity compared to T09, presumably mediated by LXR through side activity.

We found that sterols are important endogenous LXR agonists regulating ALOX15. We could show that particularly the oxysterols 24(*S*),25-Ep cholesterol, and 22(*R*)-OH cholesterol and the cholesterol precursor desmosterol modulate the ARA cascade in macrophages, presumably by LXR. Thus, our results show a new cross-link between two lipid mediator classes: sterols/oxysterols and oxylipins. Sterols affect the LXR-mediated formation of 15-LOX-derived oxylipins. This regulatory mechanism should be further investigated, as the 15-LOX-derived oxylipins are suggested to play an important role in inflammatory resolution. A detailed characterization of this regulatory pathway could provide insights into the lipid mediator switch in macrophages leading to the formation of anti-inflammatory oxylipins.

## Figures and Tables

**Table 1:** Oxylipin concentrations and 15-LOX levels in M2-like macrophages. Primary blood monocytic cells were differentiated into M2-like macrophages with 10 ng/mL M-CSF for 8 days and incubated with IL-4 for the final 48 h. M2-like macrophages were incubated with 1 µM T09 for different periods of time to investigate the time-dependent correlation of 15-LOX abundance and activity (top) and with different T09 concentrations for 24 h to determine the dose-dependent correlation (bottom). The results are shown as mean ± SEM (*n* = 3–5).

Time-Dependent Incubations [pmol/mg Protein]								
		Ctrl	3 h	6 h	18 h	24 h	30 h	48 h
Oxylipin concentration	12-HETE	15.5 ± 2.2	22.7 ± 2.2	26.4 ± 3.3	27.1 ± 3.4	32.5 ± 3.4	33.5 ± 8.9	29.3 ± 2.9
	15-HETE	184 ± 29	256 ± 40	277 ± 34	280 ± 25	327 ± 16	476 ± 213	364 ± 100
	5,15-diHETE	9.18 ± 1.1	25.6 ± 4.9	28.3 ± 2.8	33.7 ± 1.3	45.9 ± 8.2	59.6 ± 20	57.3 ± 6.4
	5,12-diHETE	0.47 ± 0.1	1.00 ± 0.4	1.23 ± 0.2	1.76 ± 0.4	2.17 ± 0.2	1.11 ± 0.4	0.64 ± 0.1
	5,15-diHEPE	3.30 ± 0.4	7.73 ± 0.4	9.60 ± 1.4	12.9 ± 1.9	14.0 ± 2.5	10.4 ± 0.9	10.2 ± 3.2
	8,15-diHETE	0.28 ± 0.1	0.55 ± 0.1	0.99 ± 0.3	0.98 ± 0.2	0.87 ± 0.2	0.83 ± 0.3	0.64 ± 0.5
	7,17-diHDHA	10.7 ± 1.0	21.1 ± 3.7	25.5 ± 0.8	33.1 ± 1.3	40.4 ± 1.7	56.9 ± 17	39.5 ± 5.3
	12-HEPE	1.60 ± 0.3	1.93 ± 0.2	2.29 ± 0.3	2.32 ± 0.1	2.11 ± 0.5	1.20 ± 0.9	1.23 ± 0.7
	15-HEPE	18.9 ± 2.9	22.1 ± 2.7	26.1 ± 3.1	28.83 ± 1.8	22.1 ± 4.1	11.7 ± 8.9	12.1 ± 8.5
	14-HDHA	16.2 ± 4.0	18.3 ± 3.3	22.3 ± 4.1	24.7 ± 2.0	25.0 ± 7.5	14.2 ± 12	14.4 ± 14
	7-HDHA	3.65 ± 1.6	2.46 ± 0.8	2.89 ± 1.0	2.86 ± 0.4	2.98 ± 1.1	2.13 ± 1.8	2.18 ± 1.5
	17-HDHA	45.4 ± 14	40.3 ± 8.5	51.4 ± 13	52.2 ± 5.9	50.7 ± 15	33.3 ± 29	34.0 ± 39
	15-LOX level	2.95 ± 0.6	4.40 ± 0.9	4.81 ± 0.6	5.66 ± 1.1	5.95 ± 0.8	2.04 ± 1.0	1.76 ± 0.6
**Dose-Dependent Incubations [pmol/mg Protein]**								
		Ctrl	0.3 µM	1 µM	3 µM	10 µM		
Oxylipin concentration	12-HETE	2.78 ± 1.0	5.55 ± 2.1	6.86 ± 2.9	7.53 ± 2.7	10.1 ± 4.0		
	15-HETE	39.9 ± 20	66.2 ± 33	86.3 ± 31	85.3 ± 31	126 ± 53		
	5,15-diHETE	1.02 ± 0.7	1.93 ± 1.6	2.73 ± 1.3	2.10 ± 1.0	4.66 ± 3.1		
	5,12-diHETE	0.22 ± 0.1	0.20 ± 0.1	0.30 ± 0.1	0.23 ± 0.1	0.34 ± 0.2		
	5,15-diHEPE	0.37 ± 0.1	0.80 ± 0.2	1.11 ± 0.3	1.29 ± 0.5	1.68 ± 0.8		
	8,15-diHETE	0.14 ± 0.1	0.13 ± 0.1	0.20 ± 0.02	0.06 ± 0.02	0.20 ± 0.05		
	7,17-diHDHA	0.71 ± 0.4	1.79 ± 1.2	1.83 ± 0.9	2.34 ± 1.8	3.94 ± 2.6		
	12-HEPE	0.32 ± 0.14	0.80 ± 0.3	0.83 ± 0.3	0.80 ± 0.2	1.04 ± 0.3		
	15-HEPE	5.20 ± 2.7	9.29 ± 4.5	10.2 ± 3.8	9.00 ± 2.7	12.4 ± 4.4		
	14-HDHA	1.79 ± 0.8	4.51 ± 2.1	4.83 ± 2.1	5.43 ± 1.9	6.07 ± 2.2		
	7-HDHA	0.88 ± 0.5	1.63 ± 1.0	1.97 ± 0.9	1.60 ± 0.5	1.50 ± 0.6		
	17-HDHA	6.32 ± 3.4	14.4 ± 8.4	15.5 ± 6.2	15.4 ± 5.3	18.4 ± 7.1		
	15-LOX level	0.77 ± 0.1	1.00 ± 0.2	1.29 ± 0.2	1.22 ± 0.1	1.13 ± 0.1		

## Data Availability

Data are contained within the article and Supplementary Materials.

## Supplementary Materials

The following supporting information can be downloaded at: https://www.mdpi.com/article/10.3390/molecules29081745/s1, Table S1: Selection of iNOS peptides from an in silico tryptic digest; Table S2: Selected proteotypic peptides (PTPs) for LXRα, LXRβ, PPARγ, IL-1RII, TLR2, TLR4, and iNOS; Table S3: Selected transitions for (A) unlabeled and (B) heavy labeled (lys: U-^13^C_6_; U-^15^N_2_; arg: U-^13^C_6_; U-^15^N_4_) peptide data for LXRα, LXRβ, PPARγ, IL-1RII, TLR2, TLR4, iNOS; Table S4: Oxylipin concentrations and 15-LOX levels in M2-like macrophages induced by LXR, FXR, and RXR agonists and cholesterol precursors and metabolites; Figure S1: Immunostaining of macrophage surface markers; Figure S2: Effect of the LXR agonist T09 on the monohydroxylated oxylipin formation; Figure S3: Time- and dose-dependent 15-LOX activity; Figure S4: 15-LOX activity induced by LXR, FXR, and RXR agonists and identification of cholesterol precursors and metabolites; Figure S5: Cell viability assay of the test compounds in M2-like macrophages. Refs. [85,86,87,88,89,90,91,92,93,94,95,96] are cited in Supplementary Materials.
