# Supplementary material for: Sterol Derivatives Specifically Increase Anti-Inflammatory Oxylipin Formation in M2-like Macrophages by LXR-Mediated Induction of 15-LOX
Source: Molecules. 2024 Apr 12;29(8):1745. doi: 10.3390/molecules29081745 (PMC11052137; doi:10.3390/molecules29081745)
Supplement: Supplementary file 1 [file molecules-29-01745-s001.zip › molecules-2909036-supplementary.pdf]

Sterol derivatives specifically increase anti-inflammatory oxylipin formation in M2-like macrophages by LXR mediated induction of 15-LOX

Reiichi Ohno†, Malwina Mainka†, Rebecca Kirchhoff, Nicole M. Hartung, Nils Helge Schebb *

Chair of Food Chemistry, Faculty of Mathematics and Natural Sciences, University of Wuppertal, Gaußstr. 20, 42119 Wuppertal, Germany

† These authors contributed equally to this work.

* Corresponding author (Tel: +49 202-439-3457; E-mail: nils@schebb-web.de)

Supplementary Materials

Contents

Selection of peptides 3

Table S1: Selection of iNOS peptides from *in silico* tryptic digest. 5

Table S2: Selected proteotypic peptides (PTPs) for LXRα, LXRβ, PPARγ, IL-1RII, TLR2, TLR4, iNOS. 6

Table S3: Selected transitions for (A) unlabeled and (B) heavy labeled (lys: U-13C6; U-15N2; arg: U-13C6; U-15N4) peptide data for LXRα, LXRβ, PPARγ, IL-1RII, TLR2, TLR4, iNOS. 7

Table S4: Oxylipin concentrations and 15-LOX levels in M2-like macrophages induced by 9

Figure S1: Immunostaining of macrophage surface markers. 10

Figure S2 11

Figure S3: Time- and dose-dependent 15-LOX activity. 13

Figure S4: 15-LOX activity induced by LXR, FXR and RXR agonists and identification of cholesterol precursors and metabolites 14

Figure S5: Cell viability assay of the test compounds in M2-like macrophages. 15

Selection of peptides

For analysis of proteins using a quantitative targeted proteomics method, unique proteotypic peptides (PTPs) [85] are selected to ensure that the target protein is unambiguously identified and can be detected.

The following human proteins were selected to characterize macrophage differentiation and to quantify LXR:

- Oxysterols receptor LXR-alpha (LXRα; Uniprot accession no.: Q13133)
- Oxysterols receptor LXR-beta (LXRβ; Uniprot accession no.: P55055)
- Peroxisome proliferator-activated receptor gamma (PPARγ; Uniprot accession no.: P37231)
- Interleukin-1 receptor type 2 (IL-1RII; Uniprot accession no.: P27930)
- Toll-like receptor 2 (TLR2; Uniprot accession no.: O60603)
- Toll-like receptor 4 (TLR4; Uniprot accession no.: O00206)
- Inducible Nitric oxide synthase (iNOS; Uniprot accession no.: P35228)

The detailed procedure is described here exemplarily iNOS (gene: NOS2). The petides for the other proteins were selected accordingly.

An *in silico* tryptic digestion using ExPASy peptide mass and peptide cutter [86] was carried out, whereby 128 peptides with lengths between 2 and 43 amino acids (aa) were initially obtained. These peptides were analyzed for their suitability as PTPs based on certain criteria. Peptides shorter than 7 (low probability of unique sequence) and longer than 22 aa (poor MS ionization or out of measuring range) were discarded [83] and 58 peptides remained (Table S1).

These peptides were checked for uniqueness in the human proteome using the NextProt peptide uniqueness checker [87] and NCBI Blast [88] databases. Two of the checked peptides were not unique, as they are also found in the sequence of other proteins. The theoretical cleavage probability for the C-terminal cleavage site of the peptides was calculated using ExPASy peptide cutter [86] and additionally the overall cleavage probability was predicted using the decision tree CP-DP [89]. Peptides with a cleavage probability <90% or a total cleavage probability <70% were excluded, leaving 48 peptides.

The remaining peptides were screened for posttranslational modifications (PTMs) (Uniprot [90], Phosphosite Plus [91]), such as phosphorylation or glycosylation, or

naturally occurring variants resulting from single nucleotide polymorphisms (SNPs) [90]. Alternative splice variants of the corresponding protein were also considered [90] and 33 peptides remained. Furthermore, the presence of certain aa (cysteine (C), asparagine (N) and glutamine (Q, especially N-terminal), methionine (M), tryptophan (W)) may be probe to chemical modifications [92-94], such that a maximum of two of these aa are tolerated in a peptide. Finally, 18 peptides were left.

In order to also ensure that the selected peptides elute within an optimal detection window of the chromatographic method, an estimated retention time was calculated using Sequence Specific Retention Calculator (SSRCalc [95]; gradient delay time: 1.5 min, acetonitrile gradient: 1.05 %B/min, "100 Å C18 column, 0.1% Formic Acid 2015"). After these calculations, 4 peptides remained (Table S2) and they were ordered as standards as well as corresponding heavy labeled peptides were obtained from JPT Peptides (Berlin, Germany).

For the selected peptides, product ion spectra were recorded and 3-5 of the most intensive fragments per peptide were selected to achieve the highest possible sensitivity. The SRMAtlas database [96] was used to assist the characterization of the fragments. The collision energy was optimized for all transitions. Constant values were used for all other parameters, e.g. 40 V for the declustering potential, which were optimized for the detection of peptides [83]. The same fragments and potentials were selected for both unlabeled and heavy labeled peptides with the terminal arginine and lysine being uniformly labeled by ^{13}C and ^{15}N isotopes.

After optimization of the transitions, the peptides were spiked into primary M0 macrophage lysates at a concentration of 50 nM. Two of the selected peptides showed poor sensitivity and matrix interference. Finally, for the two remaining peptides, two transitions (one quantifier and one qualifier) each, which are particularly sensitive and have only few matrix interferences, were selected (Table S3). In addition, the ratio between the two transitions is monitored to ensure peak identity and thus reliable quantification of the peptides in the samples.

Table S1: Selection of iNOS peptides from *in silico* tryptic digest. Selection of peptides was based on peptide length (7-22 aa), uniqueness, cleavage probability and prediction, known posttranslational modifications (PTMs), single nucleotide polymorphism (SNPs), number of unfavored aa and predicted retention time (tR). Shown are only peptides with lengths between 7-22 aa. Peptides selected for the method are shown in bold.

peptide sequence	position	$[M+H]^+$	peptide length [aa]	uniqueness [1)]	C-terminal cleavage probability [%] [2)]	overall cleavage probability [%] [3)]	SNPs [4)]	PTM [5)]	unfavoured aa [6)]	predicted tR [min] [7)]
DPELFEIPPDLVLEVAMEHPK	315-335	2418.2	21	unique	100	84			1 x M	-
APCATSSPVTQDDLQYHNLSK	31-51	2275.1	21	unique	100	88		S37: p; S50: p	1 x N; 2 x Q; 1 x C	-
EIETTGTYQLTGDELIFATK	172-191	2230.1	20	unique	100	86	177:G → V		1 x Q	-
LLLVVTSTFGNGDCPGNGEK	585-604	2021.0	20	unique	95	89			2 x N; 1 x C	-
SEALAWDLGALFSCAFNPK	550-568	2040.0	19	unique	100	87	552:A → G		1 x N; 1 x W; 1 x C	-
DHTPTEIHLTVAVVTYHTR	915-933	2190.1	19	unique	100	94	933:R → G			-
GDPANVEFTQLCIDLGWKPK	279-298	2231.1	18	unique	100	12			1 x N; 1 x Q; 1 x W; 1 x C	-
FTNSPTFLEVLEEFPSLR	873-890	2126.1	18	unique	100	92			1 x N	30.0
QNVTIMDHHSAAESFMK	429-445	1945.9	17	unique	100	93	439:A → T		2 x M; 1 x N; 1 x Q	-
DQAVVEINIAVLHSFQK	412-428	1911.0	17	unique	100	90	423:L → I		1 x N; 2 x Q	-
YHEDIFGAVFPYEAK	1123-1137	1785.8	15	unique	91	81				18.8
NWGSGMTFQDTLHHK	89-103	1758.8	15	unique	100	87			1 x M; 1 x N; 1 x Q; 1 x W	-
LEALDESGSYWVSDK	803-817	1698.8	15	unique	74	77	805:A → D		1 x W	-
LNEEQVEDYFFQLK	1105-1118	1801.9	14	unique	100	79			1 x N; 2 x Q	11.1
LYTSNVTWDPHHYR	697-710	1788.8	14	unique	100	90			1 x N; 1 x W	-
VSAGFLLSQLPILKPR	891-906	1739.1	14	unique	50	17		S892: p	1 x Q	-
QQNESPQPLVETGK	52-65	1554.8	14	unique	93	84			1 x N; 3 x Q	-
YAVFGLGSSMYPR	621-633	1447.7	13	unique	100	94			1 x M	17.6
FDVVPLVLQANGR	302-314	1427.8	13	unique	100	86			1 x N; 1 x Q	19.5
VAVQPSSLEMSAL	1141-1153	1331.7	13	unique	-	-			1 x M; 1 x Q	19.8
IQWSNLQVFDAR	204-215	1476.8	12	unique	100	94			1 x N; 2 x Q; 1 x W	-
LEALCQPSEYSK	859-870	1367.7	12	unique	100	77			1 x Q; 1 x C	-
YAGYQMPDGSIR	267-278	1357.6	12	unique	100	93			1 x M; 1 x Q	10.9
EPGHLYVCGDVR	1075-1086	1344.6	12	unique	100	75			1 x C	-
LVQDSQPLDLSK	711-722	1342.7	12	unique	100	90			2 x Q	12.3
GVLHAVHTAYSR	1036-1047	1310.7	12	unique	100	87				7.7
VVDGPTPHQTVR	791-802	1305.7	12	unique	100	93	800:T → A		1 x Q	5.2
FCAFAHDIDQK	634-644	1294.6	11	unique	100	91			1 x Q; 1 x C	-
LAQVATEEPER	846-856	1242.6	11	unique	100	92			1 x Q	7.5
**VTILFATETGK**	**539-549**	**1179.7**	**11**	**unique**	**91**	**87**				**15.3**
FHQYAMNGEK	13-22	1224.5	10	unique	83	78			1 x M; 1 x N; 1 x Q	-
AVLFACMLMR	521-530	1154.6	10	unique	100	84			2 x M; 1 x C	-
QNLQSPTSSR	741-750	1117.6	10	unique	92	86	747:T → A	S745: p	1 x N; 2 x Q	-
LDATPLSSPR	74-83	1056.6	10	unique	100	85		T77: p; S81: p		-
SCLGSIMTPK	114-123	1036.5	10	unique	100	76			1 x M; 1 x C	-
QQLASEVLR	1062-1070	1043.6	9	unique	100	95			2 x Q	-
**YNILEEVGR**	**389-397**	**1092.6**	**9**	**unique**	**96**	**93**			**1 x N**	**15.1**
YSTNNGNIR	233-241	1038.5	9	unique	100	94		S234: p	3 x N	-
SAITVFPQR	242-250	1018.6	9	P29474; P35228	100	88			1 x Q	-
AACETFDVR	679-687	1011.5	9	unique	100	94	679:A → S		1 x C	-
THVWQDEK	498-505	1042.5	8	unique	92	80			1 x Q; 1 x W	5.5
EMFEHICR	222-229	1064.5	8	unique	100	87			1 x M; 1 x C	-
ALSSMHAK	723-730	844.4	8	unique	88	89			1 x M	3.3
VYVQDILR	1054-1061	1005.6	8	unique	100	85		Y1055: p	1 x Q	-
VWNAQLIR	259-266	999.6	8	unique	100	94	266:R → H		1 x N; 1 x Q; 1 x W	-
LSCLEEER	577-584	978.5	8	unique	100	87		S578: p	1 x C	-
SWAVQTFK	671-678	966.5	8	unique	86	97	676:T → I		1 x Q; 1 x W	-
FYSISSSR	907-914	946.5	8	unique	84	90	913:S → P	Y1055: p	x C	-
DINNNVEK	23-30	945.5	8	unique	94	81	23:D → G		3 x N	-
MTLVFGCR	1010-1017	926.5	8	P29474; P35228	91	87			1 x M; 1 x C	-
DFCDVQR	382-388	882.4	7	unique	100	95			1 x Q; 1 x C	-
IEEHLAR	159-165	867.5	7	unique	100	89				4.3
LHDSQHK	997-1003	864.4	7	unique	100	85			1 x Q	3.4
QHIQIPK	690-696	863.5	7	unique	100	86			2 x Q	-
YMQNEYR	446-452	1003.4	7	unique	100	92			1 x M; 1 x N; 1 x Q	-
MGLETHK	399-405	815.4	7	unique	100	86			1 x M	3.3
DVAHTLK	1090-1096	783.4	7	unique	100	86				3.9
SPESLVK	67-73	759.4	7	unique	100	73				6.4

[1)]from BLAST [88] and NeXtprot [87]; [2)]calculated from peptide cutter [86] (≥ 90%); [3)]calculated from CP-DT [89] (≥ 70%); [4)]SNPs from Uniprot [90]; [5)] PTMs from Uniprot [90] and Phosphosite Plus [91] (p – phosphorylation); [6)]unfavored aa (C, M, N, Q, W; max. 2); [7)]predicted retention time (tR) from SSRCalc [95] (3 – 30 min); -: not evaluated

Table S2: Selected proteotypic peptides (PTPs) for LXRα, LXRβ, PPARγ, IL-1RII, TLR2, TLR4, iNOS. The selection is based on peptide length (7-22 aa), uniqueness, cleavage probability or prediction, occurrence of single nucleotide polymorphisms (SNPs), variation in splice variants or posttranslational modifications (PTMs), unfavored amino acids and predicted retention time (Table S1).

peptides	position	$[M+H]^+$	length [aa]	uniqueness 1)	C-terminal cleavage probability 2) [%]	overall cleavage probability 3) [%]	SNPs 4)	variation in splice variants 5)	PTMs 6)	unfavored aa 7)	pred. tR 8) [min]
**Oxysterols receptor LXR-alpha (LXRα; Q13133; gene: NR1H3)**											
EECVLSEEQIR *	165-175	1334.6	11	unique	100	86	-	-	-	1 x Q	12.0
MLGNELCSVCGDK *	92-104	1368.6	13	unique	93	83	-	-	-	1 x M; 1 x N	12.6
TSAIEVMLLETSR	292-304	1449.8	13	unique	86	95	-	-	-	1 x M	20.1
**Oxysterols receptor LXR-alpha (LXRα; Q13133; gene: NR1H3) / Oxysterols receptor LXR-beta (LXRβ; P55055; gene: NR1H2)**											
TLSSVHSEQVFALR	416-429	1573.8	14	no	100	88	-	-	-	1 x Q	13.8
**Oxysterols receptor LXR-beta (LXRβ; P55055; gene: NR1H2)**											
VEALQQPYVEALLSYTR	391-407	1980.0	17	unique	100	77	-	-	-	2 x Q	21.6
VTPWPLGADPQSR	248-260	1423.7	13	unique	84	87	-	-	-	1 x W; 1 x Q	13.7
YNHETECITFLK	319-330	1497.7	12	unique	86	92	-	-	-	1 x N; 1 x H	10.4
**Peroxisome proliferator-activated receptor gamma (PPARγ; P37231, gene: PPARG)**											
LNHPESSQLFAK	451-462	1370.7	12	unique	95	93	-	missing in isoform 3	-	1 x N; 1 x Q; 1 x H	9.4
HLYDSYIK	245-252	1038.5	8	unique	100	92	-	missing in isoform 3	-	1 x H	9.1
TDPVVADYK	86-94	1007.5	9	unique	100	90	-	-	-	-	9.2
**Interleukin-1 receptor type 2 (IL-1RII; P27930; gene: IL1R2)**											
EDLHMDFK	318-325	1034.5	8	unique	100	90	-	missing in isoform 2	-	1 x M; 1 x H	9.8
TVPGEEETR	77-85	1017.5	9	unique	100	91	-	-	-	-	4.2
TISASLGSR	245-253	891.5	9	unique	100	86	-	-	-	-	7.9
**Toll-like receptor 2 (TLR2; O60603; gene: TLR2)**											
TVFVLSENFVK	699-709	1282.7	11	unique	100	87	-	-	-	1 x N	19.3
EQLDSFHTLK	517-526	1217.6	10	unique	100	92	-	-	-	1 x Q; 1 x H	11.4
VGNMDTFTK	156-164	1012.5	9	unique	100	90	-	-	-	1 x N; 1 x M	9.1
**Toll-like receptor 4 (TLR4; O00206; gene: TLR4)**											
DFIPGVAIAANIIHEGFHK	711-729	2049.1	19	unique	100	77	-	-	-	1 x N; 2 x H	21.6
NLDLSFNPLR	58-67	1188.6	10	unique	100	92	-	-	-	2 x N	17.4
LVLGEFR	258-264	833.5	7	unique	100	90	-	-	-	-	13.4
**Nitric oxide synthase, inducible (iNOS; P35228; gene: NOS2)**											
VTILFATETGK	539-549	1179.7	11	unique	91	87	-	-	-		15.3
YNILEEVGR	389-397	1092.6	9	unique	86	92	-	-	-	1 x N	15.1

1)from BLAST [88] and NeXtprot [87]; 2)calculated from peptide cutter [86] (≥ 90%); 3)calculated from CP-DT [89] (≥ 70%); 4)SNPs from Uniprot [90]; 5) splice variants from Uniprot; 6) PTMs from Uniprot [90] and Phosphosite Plus [91] (ac – acetylation, gl – glycosylation, n – nitrosylation, p – phosphorylation); 7)unfavored aa; 8)predicted retention time (tR) from SSRCalc [95] (3 – 30 min)

- not evaluated

* carbamidomethylated C

Table S3: Selected transitions for (A) unlabeled and (B) heavy labeled (lys: U-13C6; U-15N2; arg: U-13C6; U-15N4) peptide data for LXRα, LXRβ, PPARγ, IL-1RII, TLR2, TLR4, iNOS. Shown are Q1 and Q3 *m/z*, optimized collision energies (CE), area ratios of qualifier to quantifier transitions and retention time (tR, mean ± range, within one analytical batch, n = 20)). (**A**) Linear calibration range, limit of detection (LOD) and limit of quantification (LLOQ), as well as the transitions of the corresponding internal standards (IS) are shown for quantifier transitions (**bold**). Accuracy of calibrators was within a range of ± 15% (20% for LLOQ). (**B**) The heavy labeled peptides are spiked at a concentration of 25 nM for all peptides (concentration in vial).

(A)

gene / protein	peptide	transitions	Q1 *m/z*	Q3 *m/z*	tR [min]	rel. ratio to quantifier [%]	CE (V)	IS Transitions	calibration range [nM]	LOD [pM]	LLOQ [pM]
**NR1H3 / Oxysterols receptor LXR-alpha**											
	EECVLSEEQIR	**$M^{2+} \rightarrow y_7^{+}$**	696.33	874.46	12.04 ± 0.08		35	$M^{2+} \rightarrow y_7^{+}$	0.08 - 1060	50	80
		$M^{2+} \rightarrow y_6^{+}$	696.33	761.38		82	35				
	MLGNELCSVCGDK	**$M^{2+} \rightarrow y_6^{+}$**	741.82	665.29	13.38 ± 0.12		40	$M^{2+} \rightarrow y_6^{+}$	0.45 - 897	230	450
		$M^{2+} \rightarrow y_7^{+}$	741.82	825.32		47	40				
	TSAIEVMLLETSR	**$M^{2+} \rightarrow y_7^{+}$**	725.39	849.45	21.41 ± 0.15		35	$M^{2+} \rightarrow y_7^{+}$	0.87 - 1167	530	870
		$M^{2+} \rightarrow y_5^{+}$	725.39	605.20		47	30				
**NR1H3 / Oxysterols receptor LXR-alpha ; NR1H2 / Oxysterols receptor LXR-beta**											
	TLSSVHSEQVFALR	**$M^{3+} \rightarrow b_2^{+}$**	525.28	215.14	14.15 ± 0.07		20	$M^{3+} \rightarrow b_2^{+}$	0.45 - 1270	300	450
		$M^{3+} \rightarrow y_{12}^{+}$	525.28	680.36		20	25				
**NR1H2 / Oxysterols receptor LXR-beta**											
	VEALQQPYVEALLSYTR	**$M^{3+} \rightarrow y_5^{+}$**	660.69	639.35	23.63 ± 0.08		30	$M^{3+} \rightarrow y_5^{+}$	0.61 - 339	250	610
		$M^{3} \rightarrow y_4^{+}$	660.69	526.26		63	25				
	VTPWPLGADPQSR	**$M^{2+} \rightarrow y_4^{+}$**	712.37	487.26	16.71 ± 0.10		45	$M^{2+} \rightarrow y_4^{+}$	0.47 - 1050	280	470
		$M^{2+} \rightarrow y_9^{++}$	712.37	940.49		61	40				
	YNHETECITFLK	**$M^{3+} \rightarrow y_5^{+}$**	518.91	621.40	13.75 ± 0.06		25	$M^{3+} \rightarrow y_5^{+}$	3.0 - 1220	1800	3000
		$M^{3} \rightarrow y_6^{+}$	518.91	781.43		97	25				
**PPARG / Peroxisome proliferator-activated receptor gamma**											
	LNHPESSQLFAK	**$M^{3+} \rightarrow y_2^{+}$**	457.57	218.15	10.35 ± 0.11		30	$M^{3+} \rightarrow y_2^{+}$	2.7 - 1002	1500	2700
		$M^{3+} \rightarrow y_{11}^{++}$	457.57	629.32		43	27				
	HLYDSYIK	**$M^{3+} \rightarrow y_2^{+}$**	346.85	260.20	12.18 ± 0.08		17	$M^{3+} \rightarrow y_2^{+}$	5.7 - 1230	3000	5700
		$M^{3+} \rightarrow b_2^{+}$	346.85	251.15		58	23				
	TDPVVADYK	**$M^{2+} \rightarrow y_7^{+}$**	504.26	791.43	9.07 ± 0.05		21	$M^{2+} \rightarrow y_7^{+}$	0.03 - 1035	10	28
		$M^{2+} \rightarrow b_2^{+}$	504.26	217.08		49	21				
**IL1R2 / Interleukin-1 receptor type 2**											
	TVPGEEETR	**$M^{2+} \rightarrow y_7^{++}$**	509.25	409.19	5.42 ± 0.07		21	$M^{2+} \rightarrow y_7^{++}$	0.05 - 559	24	54
		$M^{2+} \rightarrow b_2^{+}$	509.25	201.12		10	21				
	TISASLGSR	**$M^{2+} \rightarrow y_7^{+}$**	446.25	677.36	8.54 ± 0.12		22	$M^{2+} \rightarrow y_7^{+}$	0.01 - 1110	4	11
		$M^{2+} \rightarrow y_5^{+}$	446.25	519.29		18	25				
**TLR2 / Toll-like receptor 2**											
	TVFVLSENFVK	**$M^{2+} \rightarrow y_7^{+}$**	641.86	836.45	19.35 ± 0.11		29	$M^{2+} \rightarrow y_7^{+}$	0.5 - 1054	280	500
		$M^{2+} \rightarrow b_2^{+}$	641.86	201.12		77	27				
	EQLDSFHTLK	**$M^{3+} \rightarrow y_8^{++}$**	406.54	480.76	11.49 ± 0.07		18	$M^{3+} \rightarrow y_8^{++}$	0.12 - 1200	75	120
		$M^{3+} \rightarrow y_6^{++}$	406.54	366.71		20	19				
	VGNMDTFTK	**$M^{2+} \rightarrow y_2^{+}$**	506.74	248.16	10.15 ± 0.08		34	$M^{2+} \rightarrow y_2^{+}$	0.25 - 1050	100	250
		$M^{2+} \rightarrow b_3^{+}$	506.74	271.14		88	26				
**TLR4 / Toll-like receptor 4**											
	NLDLSFNPLR	**$M^{2+} \rightarrow y_8^{+}$**	594.82	961.51	20.19 ± 0.12		26	$M^{2+} \rightarrow y_8^{+}$	0.24 - 1140	109	243
		$M^{2+} \rightarrow y_6^{+}$	594.82	733.40		62	29				
	LVLGEFR	**$M^{2+} \rightarrow b_2^{+}$**	417.25	213.16	15.48 ± 0.10		18	$M^{2+} \rightarrow b_2^{+}$	0.1 - 1080	75	98
		$M^{2+} \rightarrow y_2^{+}$	417.25	322.19		58	29				
**NOS2 / Nitric oxide synthase, inducible**											
	VTILFATETGK	**$M^{2+} \rightarrow y_8^{+}$**	590.34	866.46	16.82 ± 0.08		27	$M^{2+} \rightarrow y_8^{+}$	0.028 - 1006	15	28
		$M^{2+} \rightarrow y_9^{+}$	590.34	979.55		68	23				
	YNILEEVGR	**$M^{2+} \rightarrow y_6^{+}$**	546.79	702.38	16.57 ± 0.07		27	$M^{2+} \rightarrow y_6^{+}$	0.069 - 964	43	69
		$M^{2+} \rightarrow y_5^{+}$	546.79	589.29		73	28				

(B)

Gene / Protein	Peptide	Transitions	Q1 *m/z*	Q3 *m/z*	tR [min]	Rel. Ratio to quantifier [%]
**NR1H3 / Oxysterols receptor LXR-alpha**						
	EECVLSEEQIR	$M^{2+} \rightarrow y_7^{+}$	701.33	884.46	12.04 ± 0.08	
		$M^{2+} \rightarrow y_6^{+}$	701.33	771.38		82
	MLGNELCSVCGDK	$M^{2+} \rightarrow y_6^{+}$	745.83	673.30	13.38 ± 0.12	
		$M^{2+} \rightarrow y_7^{+}$	745.83	833.32		46
	TSAIEVMLLETSR	$M^{2+} \rightarrow y_7^{+}$	730.39	859.45	21.41 ± 0.15	
		$M^{2+} \rightarrow y_5^{+}$	730.39	615.20		44
**NR1H3 / Oxysterols receptor LXR-alpha ; NR1H2 / Oxysterols receptor LXR-beta**						
	TLSSVHSEQVFALR	$M^{3+} \rightarrow b_2^{+}$	528.62	215.14	14.15 ± 0.07	
		$M^{3+} \rightarrow y_{12}^{+}$	528.62	685.36		20
**NR1H2 / Oxysterols receptor LXR-beta**						
	VEALQQPYVEALLSYTR	$M^{3+} \rightarrow y_5^{+}$	664.02	649.35	23.63 ± 0.08	
		$M^{3} \rightarrow y_4^{+}$	664.02	536.27		71
	VTPWPLGADPQSR	$M^{2+} \rightarrow y_4^{+}$	717.37	497.27	16.71 ± 0.10	
		$M^{2+} \rightarrow y_9^{++}$	717.37	950.49		63
	YNHETECITFLK	$M^{3+} \rightarrow y_5^{+}$	521.59	629.40	13.75 ± 0.06	
		$M^{3} \rightarrow y_6^{+}$	521.59	789.43		97
**PPARG / Peroxisome proliferator-activated receptor gamma**						
	LNHPESSQLFAK	$M^{3+} \rightarrow y_2^{+}$	460.24	226.15	10.35 ± 0.11	
		$M^{3+} \rightarrow y_{11}^{++}$	460.24	633.31		57
	HLYDSYIK	$M^{3+} \rightarrow y_2^{+}$	349.51	268.20	12.18 ± 0.08	
		$M^{3+} \rightarrow b_2^{+}$	349.51	251.15		58
	TDPVVADYK	$M^{2+} \rightarrow b_2^{+}$	508.26	217.08	9.07 ± 0.05	
		$M^{2+} \rightarrow y_7^{+}$	508.26	799.43		50
**IL1R2 / Interleukin-1 receptor type 2**						
	TVPGEEETR	$M^{2+} \rightarrow b_2^{+}$	514.25	201.12	5.42 ± 0.07	
		$M^{2+} \rightarrow y_7^{++}$	514.25	414.19		11
	TISASLGSR	$M^{2+} \rightarrow y_7^{+}$	451.25	687.36	8.54 ± 0.12	
		$M^{2+} \rightarrow y_5^{+}$	451.25	529.29		10
**TLR2 / Toll-like receptor 2**						
	TVFVLSENFVK	$M^{2+} \rightarrow y_7^{+}$	645.86	844.45	19.35 ± 0.11	
		$M^{2+} \rightarrow b_2^{+}$	645.86	201.12		69
	EQLDSFHTLK	$M^{3+} \rightarrow y_8^{++}$	409.21	484.76	11.49 ± 0.07	
		$M^{3+} \rightarrow y_6^{++}$	409.21	370.71		21
	VGNMDTFTK	$M^{2+} \rightarrow y_2^{+}$	510.74	256.16	10.15 ± 0.08	
		$M^{2+} \rightarrow b_3^{+}$	510.74	271.14		86
**TLR4 / Toll-like receptor 4**						
	NLDLSFNPLR	$M^{2+} \rightarrow y_8^{+}$	599.82	971.51	20.19 ± 0.12	
		$M^{2+} \rightarrow y_6^{+}$	599.82	743.40		62
	LVLGEFR	$M^{2+} \rightarrow b_2^{+}$	422.25	213.16	15.48 ± 0.10	
		$M^{2+} \rightarrow y_2^{+}$	422.25	332.19		35
**NOS2 / Nitric oxide synthase, inducible**						
	VTILFATETGK	$M^{2+} \rightarrow y_8^{+}$	594.34	874.46	16.82 ± 0.08	
		$M^{2+} \rightarrow y_9^{+}$	594.34	987.55		67
	YNILEEVGR	$M^{2+} \rightarrow y_6^{+}$	551.79	712.38	16.57 ± 0.07	
		$M^{2+} \rightarrow y_5^{+}$	551.79	599.29		72

Table S4: Oxylipin concentrations and 15-LOX levels in M2-like macrophages induced by LXR, FXR and RXR agonists and cholesterol precursors and metabolites. Primary blood monocytic cells were differentiated to M2-like macrophages with 10 ng/mL M-CSF for 8 days and incubated with IL-4 for the final 48 h. For 24 h, M2-like macrophages were incubated with known LXR antagonist (GSK2033), FXR agonists (fexaramine and HDCA) and RXR agonists (bexarotene and 9-RA). In order to find an endogenous ligand inducing 15-LOX activity the cells were also treated with different sterols. The results are shown as mean ± SEM (n=3-5).

	Oxylipin concentrations [pmol/mg protein]									15-LOX level
	**12-HETE**	**15-HETE**	**5,15-diHETE**	**7,17-diHDHA**	**12-HEPE**	**15-HEPE**	**14-HDHA**	**7-HDHA**	**17-HDHA**	**15-LOX**
**Ctrl**	5.96 ± 2.10	85.2 ± 35.8	3.88 ± 1.87	6.10 ± 3.75	0.54 ± 0.12	7.06 ± 2.00	8.04 ± 3.47	1.95 ± 0.75	27.1 ± 11.73	0.92 ± 0.13
**GSK2033 (1 µM)**	13.6 ± 3.77	163 ± 49.3	23.4 ± 9.12	24.5 ± 18.2	1.06 ± 0.03	12.5 ± 0.56	14.4 ± 1.32	2.33 ± 0.03	38.4 ± 9.99	1.73 ± 0.82
**Fexaramine (1 µM)**	15.7 ± 8.60	206 ± 113	25.9 ± 1.19	44.9 ± 8.60	1.18 ± 0.03	14.8 ± 0.07	23.6 ± 0.43	3.54 ± 0.02	60.0 ± 3.05	1.77 ± 0.56
**HDCA (10 µM)**	4.29 ± 4.16	63.8 ± 63.2	5.49 ± 5.49	24.9 ± 24.9	0.54 ± 0.46	7.05 ± 6.82	6.30 ± 6.13	1.36 ± 1.33	22.7 ± 22.4	1.09 ± 0.79
**Bexarotene (1 µM)**	27.7 ± 8.06	309 ± 88.2	42.1 ± 18.4	49.5 ± 20.8	1.89 ± 0.01	19.1 ± 0.13	25.8 ± 0.61	4.31 ± 0.05	70.7 ± 1.67	2.25 ± 0.46
**9-RA (10 µM)**	4.15 ± 1.52	41.8 ± 21.4	0.60 ± 0.29	1.44 ± 0.81	0.54 ± 0.20	5.12 ± 2.22	4.71 ± 2.47	1.47 ± 0.88	13.7 ± 8.16	0.82 ± 0.47
**22(R)-OH Chol (10 µM)**	14.6 ±5.74	172 ± 62.6	26.2 ± 13.5	30.7 ± 14.6	1.11 ± 0.41	13.7 ± 5.15	16.9 ± 6.12	2.26 ± 0.42	43.3 ± 13.5	1.94 ± 0.20
**24(S)-OH Chol (5 µM)**	3.81 ± 2.94	47.8 ± 44.3	2.06 ± 1.99	7.76 ± 7.32	0.52 ± 0.37	6.00 ± 5.31	4.81 ± 4.43	1.18 ± 1.09	14.9 ± 14.0	0.57 ± 0.31
**25-OH Chol (10 µM)**	3.92 ± 1.78	50.3 ± 24.8	4.95 ± 3.35	6.28 ± 3.79	0.54 ± 0.22	6.49 ± 2.76	4.42 ± 1.95	1.07 ± 0.54	13.4 ± 6.06	1.10 ± 0.39
**24(S),25-Ep Chol (1 µM)**	7.26 ± 2.98	101 ± 36.6	8.77 ± 5.13	10.0 ± 5.04	0.95 ± 0.34	11.3 ± 3.81	7.81 ± 2.65	1.43 ± 0.41	22.8 ± 7.30	1.91 ±0.30
**24(S),25-Ep Chol (10 µM)**	30.8 ± 20.0	238 ± 96.9	21.9 ± 8.94	27.9 ± 10.9	1.85 ± 0.92	16.9 ± 5.27	20.7 ± 7.81	3.01 ± 1.00	55.7 ± 21.6	2.13 ± 0.40
**7-keto Chol (10 µM)**	4.27 ± 3.80	49.4 ± 47.0	4.41 ± 4.38	12.0 ± 11.7	0.50 ± 0.38	5.41 ± 4.83	5.36 ± 5.12	1.38 ± 1.32	14.7 ± 14.2	1.01 ± 0.70
**Desmosterol (1 µM)**	10.5 ± 10.4	151 ± 133	18.7 ± 24.9	136 ± 181	0.78 ± 0.69	9.47 ± 9.13	11.5 ± 11.4	1.72 ± 1.70	30.8 ± 30.5	2.68 ± 0.52

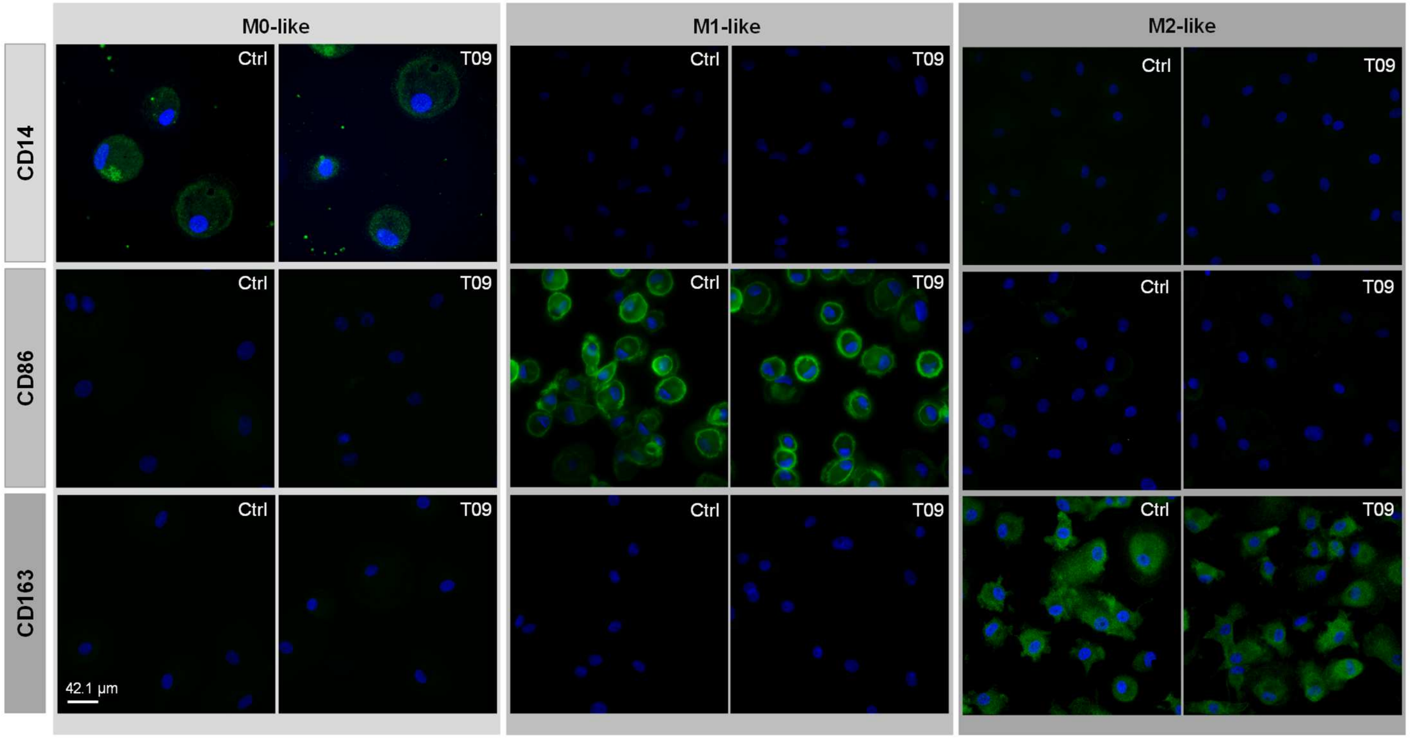

Figure S1: Immunostaining of macrophage surface markers. Primary human blood monocytic cells were treated with 10 ng/mL GM-CSF (M1 type) and M-CSF (M2 type) for 8 days as well as 10 ng/mL IFNγ (M1 type) or IL-4 (M2 type) for the final 2 days. Additionally, M1 cells were challenged with 100 ng/mL LPS for the final 6 h. For M0 type, the adhered monocytes were left untreated for 8 days. The three different macrophage phenotypes were incubated w/ or w/o the synthetic LXR agonist T09 (1 µM) for the final three hours. The three macrophage phenotypes were immunostained for specific macrophage marker CD14, CD86, or CD163 (all mouse anti-Human; Bio-Rad Laboratories, Feldkirchen, Germany) using goat anti-Mouse IgG Alexa Fluor secondary antibodies from Thermo Scientific, Langenselbold, Germany (**green**). Nuclei were counterstained with Hoechst (**blue**). Immunostaining shows that CD14 is only expressed in M0-like, CD86 in M1-like and CD 163 in M2-like macrophages. T09 has no influence on the expression of the surface markers.

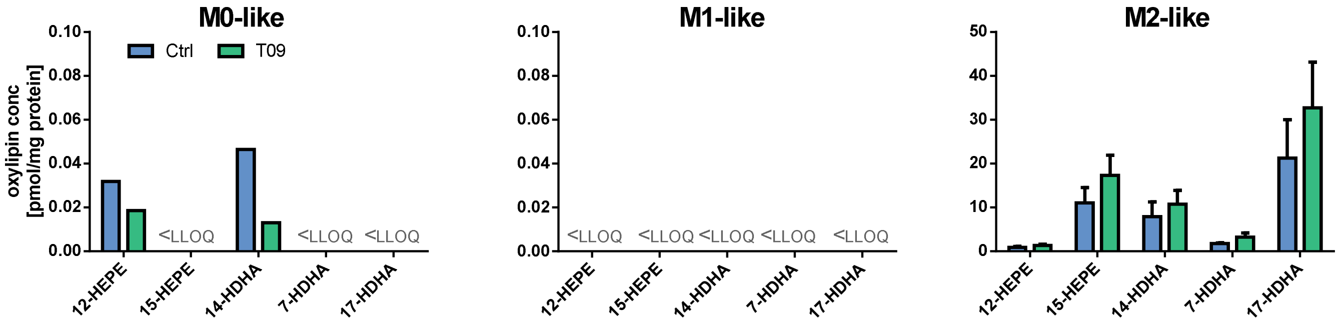

Figure S2: Effect of the LXR agonist T09 on the monohydroxylated oxylipin formation. Primary human blood monocytic cells were treated with 10 ng/mL GM-CSF (M1 type) for 8 days or with M-CSF (M2 type) for 8 days as well as 10 ng/mL IFNγ (M1 type) or IL-4 (M2 type) for the final 2 days. Additionally, M1 cells were challenged with 100 ng/mL LPS for 6 h. For M0 type, the adhered monocytes were left untreated for 8 days. The three different macrophage phenotypes were incubated w/ (**blue**) or w/o the synthetic LXR agonist T09 (1 µM; **green**) for the final three hours. Shown are monolydroxylated oxylipin concentrations of 12-HEPE and 15-HEPE (EPA-derived) and 14-HDHA, 7-HDHA and 17-HDHA (DHA-derived) (mean ± SEM; cells from 3-5 donors).

(A) time dependence (1 µM T09)

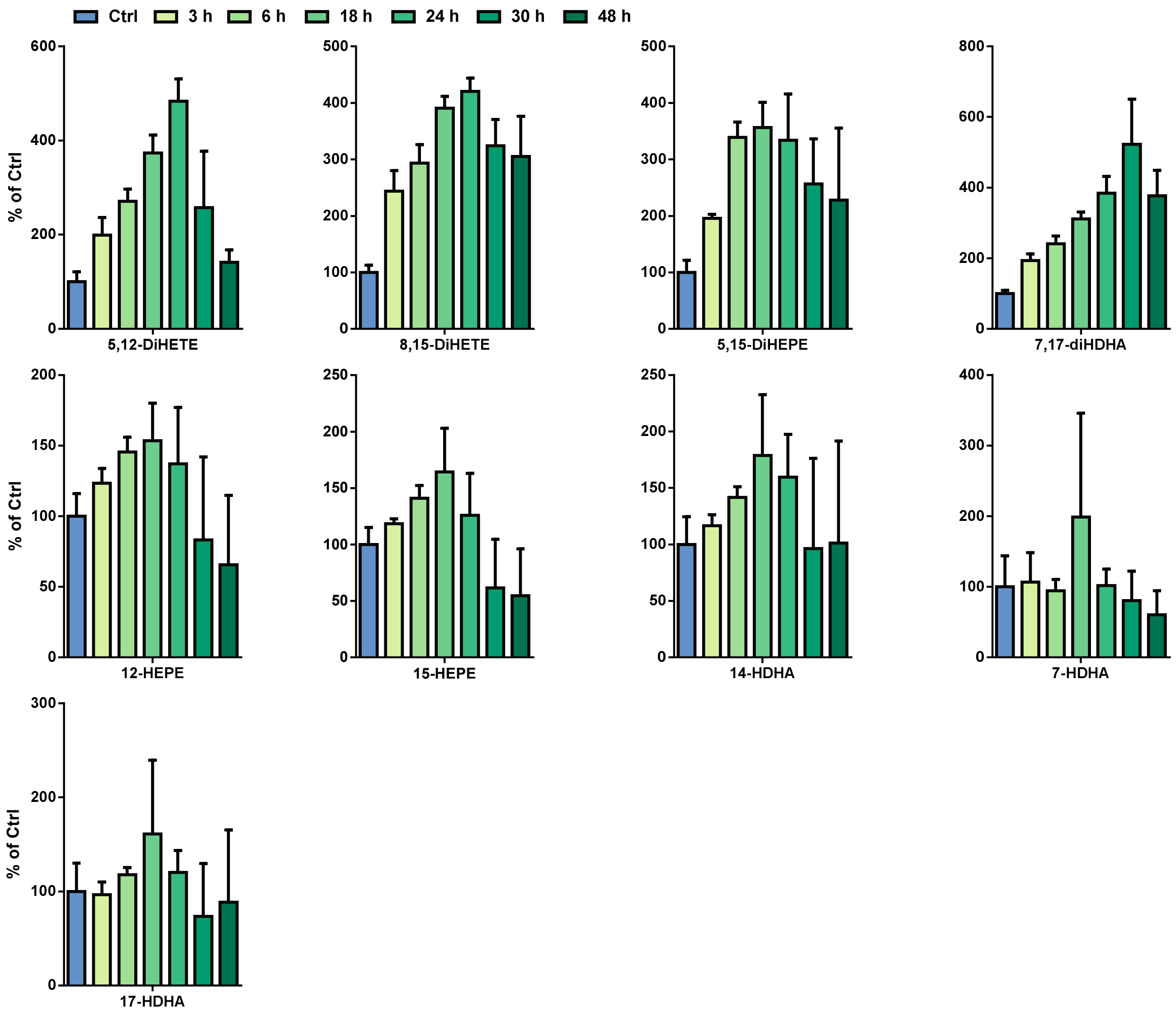

(B) dose dependence (24 h)

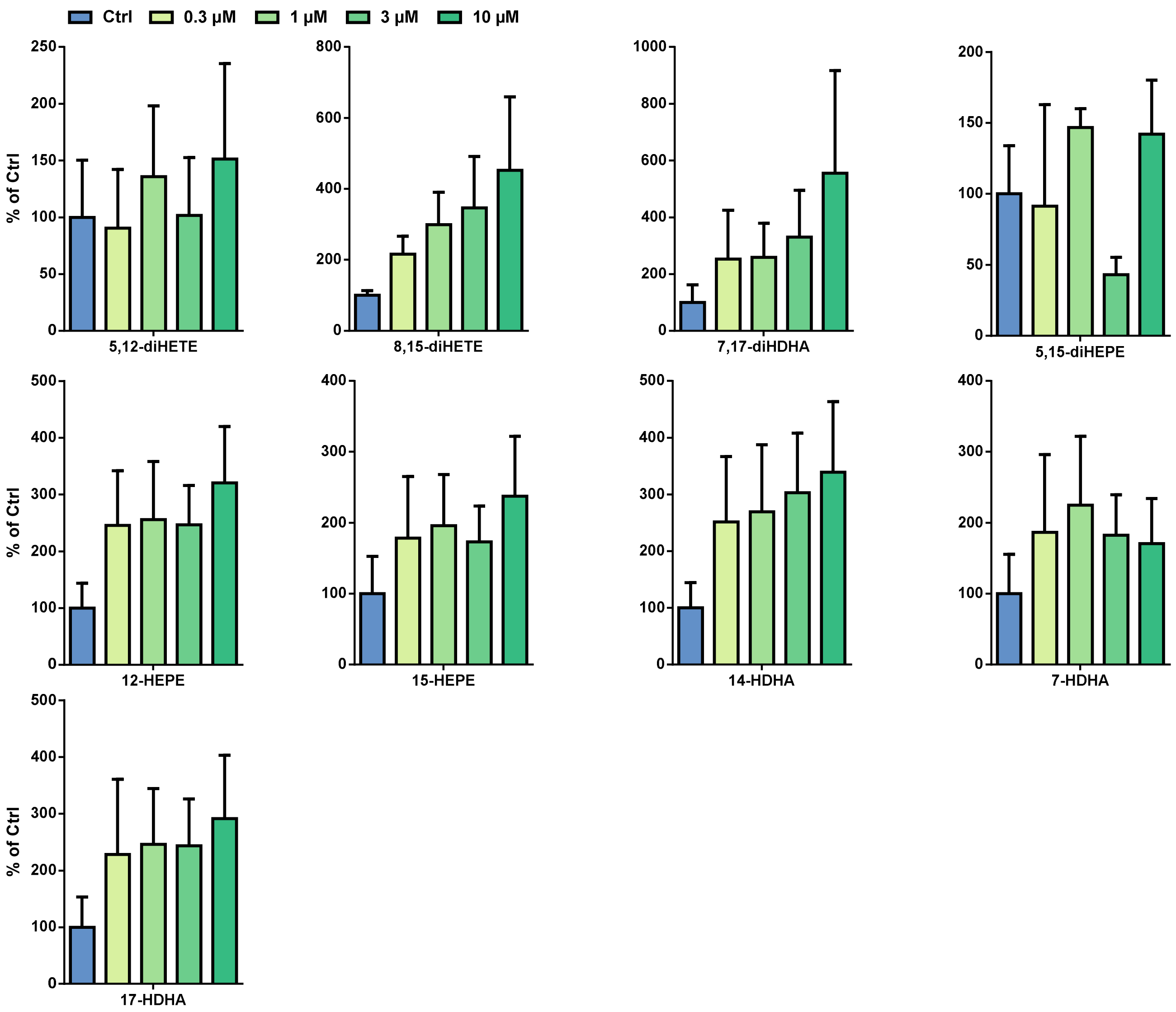

Figure S3: Time- and dose-dependent 15-LOX activity. (**A**) M2-like macrophages were incubated with 1 µM T09 for different periods of time to investigate the time-dependent of 15-LOX activity. (**B**) For the evaluation of the dose-dependence, M2-like macrophages were treated with different T09 concentrations for 24 h. Shown are relative amounts of multiple hydroxylated oxylipins 5,12-diHETE, 8,15-diHETE (ARA-derived), 5,15-diHEPE (EPA-derived) and 7,17-diHDHA (DHA-derived) and monohydroxylated oxylipins 12-HEPE and 15-HEPE (EPA-derived) and 14-HDHA, 7-HDHA and 17-HDHA (DHA-derived) (mean ± SEM, n=3-6). All incubations were repeated on two different days. Results are shown as % of Ctrl (0.1% DMSO). The concentrations determined in the cells can be found in the Table S4.

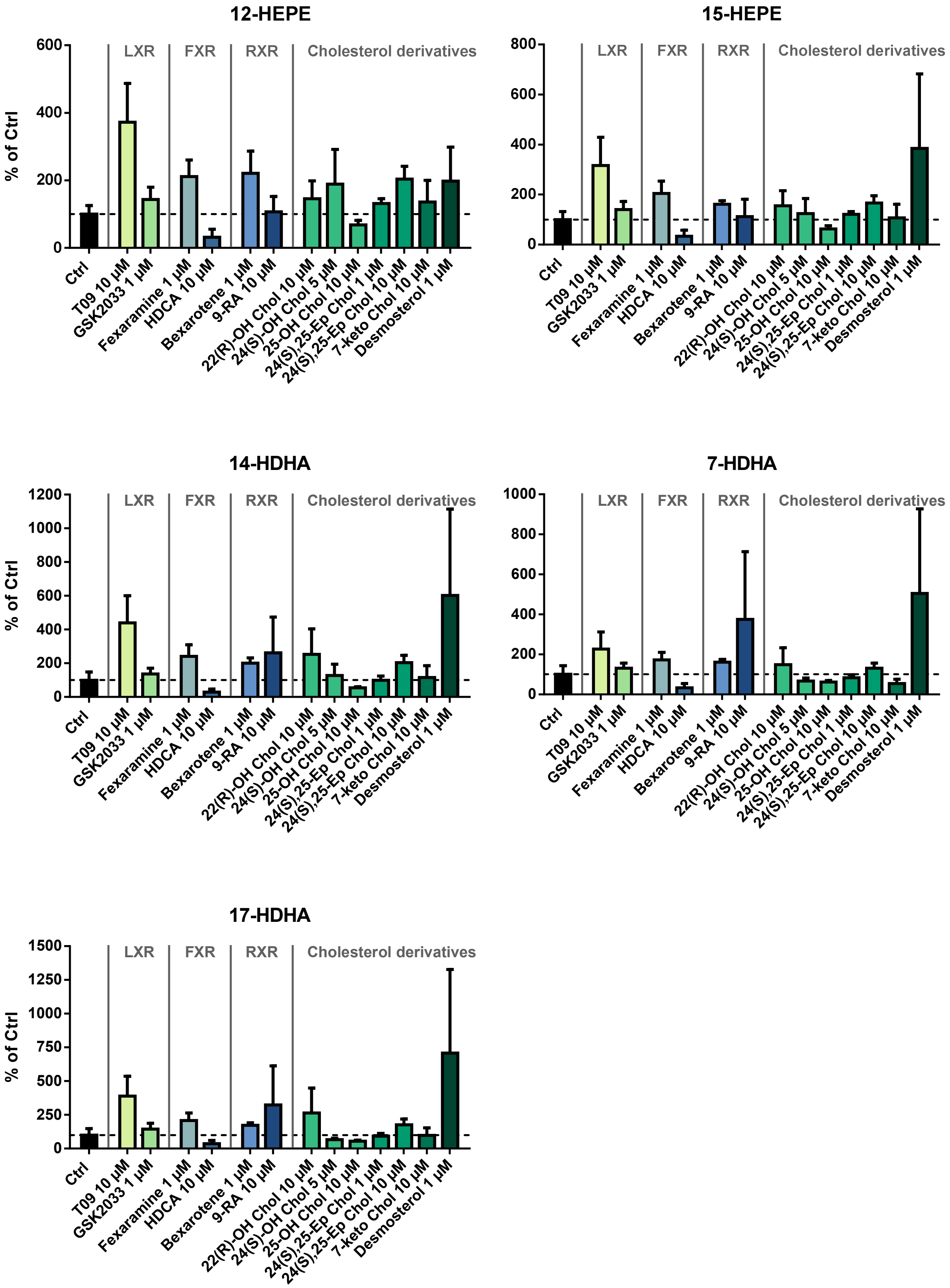

Figure S4: 15-LOX activity induced by LXR, FXR and RXR agonists and identification of cholesterol precursors and metabolites. M2-like macrophages were incubated with test compounds for 24 h. Shown is the increase of 15-LOX derived mono-hydroxylated metabolites 12-HEPE and 15-HEPE (**top**: EPA-derived) and 14-HDHA, 7-HDHA and 17-HDHA (**middle** and **bottom**: DHA-derived). Results are shown as % of Ctrl (mean ± SEM, cells from 3-5 donors).

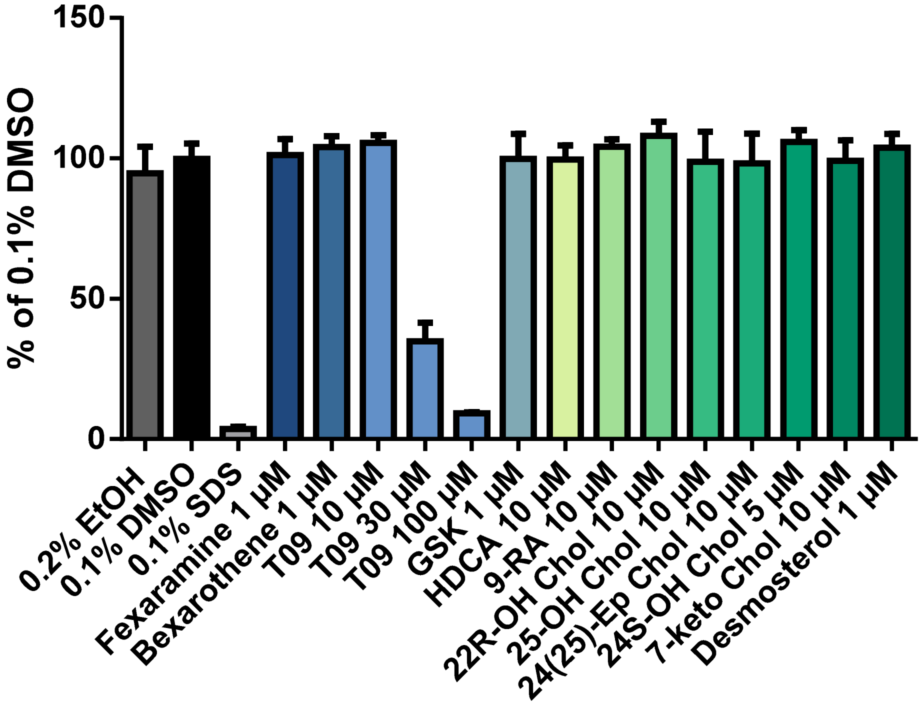

Figure S5: Cell viability assay of the test compounds in M2-like macrophages. Cell viability was determined by resazurin assay [79]. The cells were incubated with the different test compounds at the indicated concentrations for 24 h. DMSO and ethanol (EtOH) served as vehicle control and sodium dodecyl sulfate (SDS) as positive control. Dehydrogenase activity was measured as resorufin formation from resazurin (5 µg/mL) by fluorometric readout at 590 nm after excitation at 560 nm [79]. Shown are mean ± SD for n = 6-12 technical replicates from a pool of 4 donors.
